# Platelet-activating factor acetyl hydrolase IB2 dysregulated cell proliferation in ovarian cancer

**DOI:** 10.1186/s12935-021-02406-9

**Published:** 2021-12-20

**Authors:** YingYing He, Zhicheng He, Xiaoyu Zhang, Shubai Liu

**Affiliations:** 1 State Key Laboratory of Phytochemistry and Plant Resources in West China, Kunming Institute of Botany, Chinese Academy of Sciences, #132 Lanhei Road, Panlong District, Kunming, 650201 Yunnan People’s Republic of China; 2 School of Chemical Science & Technology, Yunnan University, Kunming, 650091 Yunnan China; 3 University of Chinese Academy of Sciences, Beijing, 100049 China

**Keywords:** Platelet-activating factor acetylhydrolase 1B2, Ovarian cancer, Ester lipid, Tyrosine kinase signaling pathway, Platelet-activating factor

## Abstract

**Background:**

Ovarian cancer is the leading cause of death from gynaecologic illnessed worldwide. Platelet-activating factor acetyl hydrolase IB2 (PAF-AH IB2) is an intracellular serine esterase that hydrolyzes platelet-activating factor, a G-protein-like trimer with two catalytic subunits and one regulatory subunit. The regulatory role of PAF-AH IB2 in the oncogenesis of ovarian cancer is not well understood.

**Methods:**

In this study, the TCGA dataset and clinical cancer tissue microarray were utilized to investigate abnormal overexpression of PAF-AH IB2 in ovarian cancer. To investigate the impact on the cell proliferation, migration, and tumorigenicity in vitro, PAF-AH IB2 stable knocking down (KD) ovarian cancer cells were established by ShRNA. The whole transcription profiling, tyrosine kinase profiling and standard cell functional assays were integrated to explore the biological importance and mechanism of PAF-AH IB2 modulated in ovarian cancer.

**Results:**

PAF-AH IB2 was identified significantly overexpression in four subtypes of ovarian cancer. In vitro, PAF-AH IB2 KD significantly inhibited cancer cell proliferation, migration, and tumorigenicity, activated caspases and caused cell cycle arrest, and made the cells more sensitive to PAF. PAF-AH 1B2 KD cells down-regulated several key regulators of the multiple tyrosine kinases-mediated signaling pathway, suggesting a novel interaction network between the growth factor receptors pathway and PAF-AH 1B2 mediated PAF signalling.

**Conclusions:**

These findings revealed a previously undiscovered role for PAF-AH IB2 as a potenial therapy target and essential signaling mediators in ovarian cancer pathogenesis, as well as new possible preventive and therapeutic strategies to inhibit this enzyme in clinical treatment for ovarian cancer.

**Supplementary Information:**

The online version contains supplementary material available at 10.1186/s12935-021-02406-9.

## Background

Ovarian carcer has the highest mortality rate of all gynaecological cancers. The most common epithelial ovarian malignancy, epithelial serous cancer, has a 5-year survival rate of less than 25% and a 10-year survival rate approaching zero [1]. The greater death rate of ovarian cancer is due to the later stage of disease diagnosis and lack of a viable treatment therapy approach. Therefore, developing disease-specific, target therapy approaches to improve the survival of ovarian carcer patients is critical needs. It is important to learn more about the mechanism of ovarian cancer pathogenesis and discover the key molecules that associated with malignant transformation and carcinogenesis.

Esterase enzymes are a subclass of the hydrolase enzyme superfamily that hydrolyzes ester bonds specifically [2, 3]. Different substrate specificity and biological functions have led to the identification of multiple types of esterases, and some of these enzymes have been discovered to be dysregulated and overexpressed in cancer cells [3, 4]. Metabolic pathway reprogramming, cancer etiology, drug metabolism, and drug toxicity have all been linked to esterase enzymes [2, 3]. Platelet-activating factor acetyl hydrolases (PAF-AHs) are serine esterases from phospholipase A2 family that cleave the sn-2 active side chain to hydrolyze Platelet-activating factor (PAF), which is involved in many reproductive physiology roles, such as fertilization and parturition [5, 6]. PAF-AH IB is a tissue (intracellular) type that does not share any sequence homology to other PAF-AHs in group VII. PAF is the only identified substrate of the type I PAF acetyl hydrolase in tissue [7]. PAF-AH IB is a G-protein-like trimer made up of two 29-kDa α1 (also known as PAF-AH IB3) and α2 (30 kDa) (PAF-AH IB2) catalytic subunits that form homodimers or heterodimers and share ~ 63% sequence identity, and worked as a complex with a noncatalytic 45-kDa regulatory beta subunit, LIS1 [8–10]. In humans, α2 is ubiquitously found through the body, with higher expressed in brain, kidney, spleen, etc., but pretty lower expression in heart, lung and ovarian etc. [11]. PAF-AH 1B intracellular activity was decreased in rat uterine myometrium when the protein expression changed and PAF levels increased in the later stage of pregnancy [12]. Platelet-activating factor acetyl hydrolases 1B2 and 1B3 are poorly characterized serine hydrolases that may form a protein complex with a non-catalytic protein (Lis1) to regulate the biological processes of brain development, spermatogenesis, and cancer pathogenesis [9]. Abnormal and unregulated PAF-AH 1B activity induced pregnancy-induced hypertension [13, 14]. These findings imply that PAF-AH 1B may play an important role in maintaining homeostasis through degrading PAF. PAF-AH 1B has also been identified as a potential oncogene in the oncogenesis of a variety of cancer types [15–18]. The specific role and regulated molecular mechanism of platelet activating factor-acetyl hydrolase IB act in ovarian cancer development, however, are unknown.

Here, to explore the biological importance and pathways of PAF-AH IB2 in ovarian cancer, a combination of research technologies, including the whole transcription profiling, tyrosine kinase profiling technologies as well as standard functional assays, have been used in this study. These results will aid in the defining the role of PAF-AH IB2 in ovarian cancer pathogenesis.

## Materials and Methods

### Plasmids and transfection

The human PAF-AH IB2 full length expression plasmid (pEGFP-C1-PAF-AH IB2-WT) was got as gift from Prof. Xueliang Zhu [19] and linked to co-express GFP as a marker. The mouse PAF-AH IB2 WT, functional mutants (E39D, S48C, E39D/S48C) and Lis1(pcDNA3.1-3xFlag vector) were bought from NovoPro and confirmed by sequencing. The HOSE (1 × 10^3^) cells growing in complete medium were transfected with human PAF-AH IB2 and mouse PAF-AH IB2 wild type and mutant constructs for relative functional assay.

### Explore PAF-AH IB2 gene with ovarian carcinoma cases through TCGA

The PAF-AH IB2 genes were explored in the Cancer Genomics dataset (TCGA) through Gepia and investigated the genetic alterations associated with serous ovarian carcinoma’s patient’s cases, which provides large-scale cancer patients genomics data sets from TCGA to research community for visualization, analysis and downloads [20]. Kaplan–Meier plots were generated from an online dataset (http://www.kmplot.com) [GSE15459 and GSE62254]. The disease-free survival (PFS) analysis was performed by using patient’s information. The patient’s population was split by median value.

### Ovarian cancer cell lines

The human ovarian cancer cell lines, represent the Endometrioid (Tov112D), Clear cells (RMG1), Serious (SKOV3, OVCA3, OVCA420, OVCA432, OVCA633 and OVCA 810) and Mucinous (MCAS, RMUG-L), and the normal human ovarian surface epithelial (Hose 11 and Hose 17–1) cells have been described previously [21]. These cells were bought from National collection of authenticated cell cultures (Shanghai, China) for research purpose only. HOSE cells were immortalized by an HPV E6/E7 gene introduction for research use purpose. Ovarian cancer cell lines were cultured in a medium mixture of MCDB105 medium and 199 (1:1) (Sigma, St. Louis, MO), and supplemented with 10% fetal calf serum (Invitrogen, Carlsbad, CA) and maintained in a 37 °C humidified atmosphere [95% O_2_ + 5% CO_2_].

### Whole transcriptome expression profiling

RNA was extracted from control and PAF-AH 1B2 KD cells by TRIzol reagent kit (Invitrogen, Carlsbad, CA). The RNA quality and quantity of samples were tested using spectrophotometric analysis and Bioanalyzer (Agilent Technologies, Santa Clara, CA). RNA was extracted from cell lines using TRIzol reagent (Invitrogen, Carlsbad, CA). 1  μg of RNA per each sample were used for target labelling by a two-round amplification protocol. Expression profiles were determined using 4.5 μg of fragmented, labelled and hybridized with per Chip (Human Gene whole transcript 1.1 ST Arrays, Affymetrix) The expression data were normalized by RMA pre-processing protocol, background-corrected, and log2-transformed for parametric analysis. All internal control genes were removed and the remaining probe clusters were imported into the Affymetrix Power Tools software (APT package) for next step analysis. Differentially expressed genes were identified using significance analysis of microarrays (SAM) with the R package ‘samr’ (false discovery rate (FDR) < 0.05; fold change > 2) and determining the gene list based on the number of significant genes that were identified by fold change. Two-dimensional hierarchical clusters are generated.

### Metascape pathway analysis

Gene ontology (GO) and pathway enrichment analysis of PAF-AH IB2 KD-associated significantly changed genes were performed using Metascape (http://metascape.org/) [22]. In this study, an ordered list of genes was first generated by GSEA based on correlation with PAF-AH IB2 KD. The significant survival difference observed between control and PAF-AH IB2 KD was elucidated. Gene set permutations were performed 1000 times each analysis. The nominal P-value and normalized enrichment score (NES) were used to classify the pathways enriched in each phenotype.

### Tumour tissue array immunohistochemistry analysis

Ovarian cancer tumour tissue microarray was bought from bioaitech (product ID: F100Ov01, xi’an, China), which contained formalin-fixed, paraffin-embedded normal, benign, and cancerous ovarian tissues with identified pathological diagnosis. The array included specimens of 100 ovarian malignancies of surface epithelial origin that representing five different histologic types. Sections (5 mm) were applied to detect expression of PAF-AH 1B2 in ovarian tumour tissues. Briefly, slides were deparaffinized in xylene and rehydrated by a series of graded alcohols buffers, and then 3 min boiled process in a pressure pot to retrieve the antigens. The 3% hydrogen peroxidase 10 min-treatment was used to block endogenous peroxidases. The sections were incubated with PAF-AH 1B2 antibody (20365-1-AP, Proteintech, China; overnight, 4 °C). The peroxidase conjugated secondary antibody (37 °C, 30 min) was incubated with sections and performed the chromogenic with a DAB Substrate Kit, and then counterstained with hematoxylin. The slides were then dehydrated in graded alcohol buffers and covered with coverslips. Staining intensity and percentage of PAF-AH 1B2-positive tumour cells were observed by microscope and assessed. The staining tumour tissue images were observed and evaluated by ImageJ software and IHC Profiler plugin [23]. The intensity of slide immunohistochemistry was scored automatically after the slides counting. The IHC scored values are represented as means ± SEM. The ANOVA analysis was used to compare the mean values of IHC scores between benign and different tumour histological types.

### Lentiviral knockdown and plasmid transfection

The lentiviral PAF-AH IB2-targeting and non-target control shRNA transduction particles (Mission™) were purchased from Sigma-Aldrich (St. Louis, MO). To generate stable knockdown of PAF-AH IB2, ovarian cancer cells (MCAS, SKOV3, OV432, RMGUL; 1 × 10^5^ cells) were growing in complete medium and infected with lentivirus containing pLKO short-hairpin RNA (ShRNA) constructs for PAF-AH IB2 (Sigma). After 48 h infection, cells were screened with medium containing puromycin (2 mg/ml) as the lentivirus vector contained this selection resistance marker for 2 weeks. Stable PAF-AH1b2 knockdown cell lines were validated by Western blot.

### Proliferation and scratch wound healing assay

The cell proliferation and cytotoxicity of the drugs to ovarian cancer cells were tested by tetrazolium-based MTT method [24] in time point manners. Briefly, at the beginning, the single cells solution was (5000 cells/well) allocated into each well of 96 well plates. For proliferation assay, the cells were cultured as normal, and MTT dye solution was added to each well (10 μl/well) after per 24 h cultured to incubate at 37 °C for 4 h in a humidified chamber. For drug toxicity assay, the drugs were added into plate wells after cells were completely attached. After 48 h of treatment, MTT dye solution was added into and incubated (37 °C, 4 h) in a humidified chamber. After incubation, solubilization/stop solution (100 μl/well) was added and incubated for one hour, the content of wells was mixed and read by 96-well plate scanning spectrophotometer (μQuant) and quantitative software (KC-junior, Bio-Tek Instruments, Inc.) (Absorbance value in 630 nm) for quantitative analysis. The scratch wound healing was performed using a 6 well plate. The cells were cultured for 24 h to form a confluent monolayer, then scratches were performed using a 10-μl tip and the culture medium was replaced with fresh complete medium. At the start of experiment, after 12 h, 24 h and 48 h of incubation, the plates were checked under microscope and took images to track the scratches width. All the images were converted as 8-bit images and analysed using Image J to quantitative calculate the scratches width.

### Cell proliferation, invasion and migration in real time tested by xCELLigence system

The dynamic of cell proliferation, adhesion and migration were assessed by measuring cell amount in real time manner through a xCELLigence system and E plates (Roche). It could monitor cellular events in real time through measuring electrical impedance across interdigitated gold micro-electrodes integrated on the bottom of tissue culture plates. This dynamic measurement provides quantitative data about the biological status of the cells, including cell number, viability and morphology [25]. Briefly, for determination of cell survival and proliferation, E-plate 96 (Roche Applied Science) assemblies were seeded with MCAS/SKOV3 cells (2.0 × 10^4^ cells/well). Plate was assembled on the RTCA DP analyzer, and collecting data with 5-min intervals for 20 h (37 °C, 5% CO_2_). To examine cell adhesion and migration, serum free medium was added to E-plate 16 to obtain background readings, and cells were added to wells of a CIM plate 16 (Roche Applied Science; 8-µm pore size), and dried the membranes at 25 °C for 1 h. The lower chambers were added with fresh medium (10% FBS or with serum-free medium), whereas the upper chambers were filled with serum-free medium (30 µl/well) [37 °C, 5% CO_2_, 1 h]. The cells were added to each well and balance for a while [25 °C, 30 min], then assembled the CIM plate onto the RTCA DP analyzer. The cell migration was assessed for 24 h (37 °C, 5% CO_2_) with 5-min intervals. The data were analysed using the provided RTCA software. The extent of change is proportional to the cell number, morphological and adhesive features. The more cells that are growing on the electrodes, the higher value of electrode impedance increases [25]. Cell index (CI) slope is defined to represent cell status according to the measured relative change in electrical impedance that occurs in the presence or absence of cells in the wells, which is calculated by the following formula: CI = (Zi–Z0)/15, where Zi represents the impedance at an individual time point during the experiment, and Z0 is the impedance at the start of the experiment [26].

### Colony-forming assays in soft agar gel

The scramble control and stable PAF-AH IB2 KD of MCAS Cells were cultured in soft agar gel for additional 30-day cultured followed the protocol. The cancer cells formed colonies were stained (0.5% crystal violet/20% ethanol) and taken image by light microscope. The colonies numbers were calculated by using Image J software.

### Western blot

Cells were washed with cold PBS (phosphate buffered saline) for twice, and the cellular lysates were prepared in ice-precold lysis buffer (10 mM Tris–HCl pH 7.4, 150 mM NaCl, 1 mM Na_3_VO_4_, 2 mM EDTA, 2 mM EGTA, 50 mM NaF, 1% NP-40, 1% sodium deoxycholate, 0.1% SDS, 0.5 mM DL-dithiothreitol and proteinase inhibitor cocktails, Pierce), and homogenized using a Sonic Dismembrator 100 (Fisher Scientific Inc., MA). The cell lysates protein concentration was determined by MicroBCA kit (Pierce) and equal amount of total proteins from different cell lysates were resolved by SDS-PAGE (4–12%) for Western blotting with antibodies against PAF-AH 1B1/B2/B3, beta-actin, phosphorylated p44/42 (Thr202/Tyr204), phosphorylated Akt (Ser473), phosphorylated p53 (Ser15), phosphorylated p21 Waf1, phosphor-Chk2 (Thr68), phosphorylated-CDC2 (Tyr15) and CDC (Cell Signaling, CA). Primary antibodies were visualized by secondary antibodies of goat-anti mouse (IRDye 680CW) or goat-anti rabbit (IRDye 800CW) through an Odyssey scanner (Li-cor biosciences).

### Luminex tyrosine kinases assay

The total tyrosine kinases profiles of target cells, including 62 of the 90 tyrosine kinases in the human genome, were performed by Luminex xMAP microspheres (Luminex Corporation, Austin, TX) system, which was coupled individual bead-type of antibody to capture target. According to manufacturer’s recommended procedure, each bead-type of Luminex xMAP microspheres (100 µl, Luminex Corporation, Austin, TX) were coupled separately to antibodies and performed the assays as previously described [27]. Briefly, test data were acquired through a Luminex FlexMAP 3D instrument (Luminex Corporation). The background readings value for each capture antibody were normalized by microspheres with 1 × cell lysis buffer (Cell Signaling Technology). Reading values were defined as positive only that higher threefold over the background. The results were normalized against unstimulated EGFR and presented as a fold change in relative phosphorylation. Final average results were generated from three independent experiments.

### Flow cytometry analysis

Samples were measured by BrdU-488/PI through flow cytometry (Accuri C6 Biosciences) for cell apoptosis analysis. The cells were stained exactly as recommended by the manufacturer of the Annexin kit (Promega, MA). Briefly, cells (5–10 × 10^4^) were cultured and labelled in the anoxic treatment groups and the normal oxygen groups in their medium. The cells were washed with PBS, and incubated with serum free medium for the desired times. Then, the cells were harvested with trypsin solution and washed twice with PBS. BrdU-488/PI were added into the tube and gently mixed with cells in dark condition [room temperature, 10 min]. Stained cells were washed 3 times with cold PBS and fixed with then permeabilized with 0.5% Triton X-100 in PBS [5 min, room temperature]. Finally, cells were analysed by using the flow cytometer and collected data for result analysis.

### Immunofluorescence analysis

The cells were seeded in a Chamber Slides (Nalge Nunc International) and normally cultured overnight. For the Hose cells were transfected with GFP-plasmid, cells were observed by microscopy after 24 h. Cells were treated with drugs (PAF, C-PAF, ET-18) for 24 hours and then washed twice with PBS. FITC-VAD-fmk (CaspACE™ FITC-VAD-FMK in Situ Marker, Promega) was used to test caspases activation in cells, which is a cell-permeant fluorochrome derivative of caspase inhibitor Val-Ala-DL-Asp-fluoromethylketone. Cells were washed twice by PBS and FITC-VAD-fmk (5 mM) was incubated with cells (20 min, room temperature) in the dark. Immediately after FITC-VAD-fmk staining procedure (see above), cell was co-stained with Hoechst 33342 (1 mg/ml, 10 min) for counterstaining of nuclei in the dark. Then, washing twice in PBS, cells were fixed with 0.5% paraformaldehyde (20 min, RT) in the dark. PBS washed twice and cells were resuspended in Vectashield H-100 mounting medium (Vector Laboratories, Burlingame, CA). Cells were blocked overnight at 4 °C with blocking buffer (0.1% Triton X-100, 2% BSA in PBS). The Annexin V staining to detect the cell apoptosis was followed the related protocol. Images were visualized using Zeiss Axiovert 200 inverted fluorescence microscope (40 × oil objectives) equipped with 14-bit ECCD camera and argon and krypton gas excitation asters at 488 and 568 nm. Z-stack acquisition using optimal slice distancing was performed on each microscope image.

### Statistical analysis

Significance of differences for the associations between cytotoxicity and enzyme activity, pathway activation status and metabolite profile will be determined using ANOVA with Prism software (GraphPad Software, Inc. San Diego, CA). Significance of the test was defined (i.e., *P*-value ≤ 0.05).

## Results

### Characterized the pathological role of PAF-AH 1B2 in ovarian cancer

**Fig. 1:**
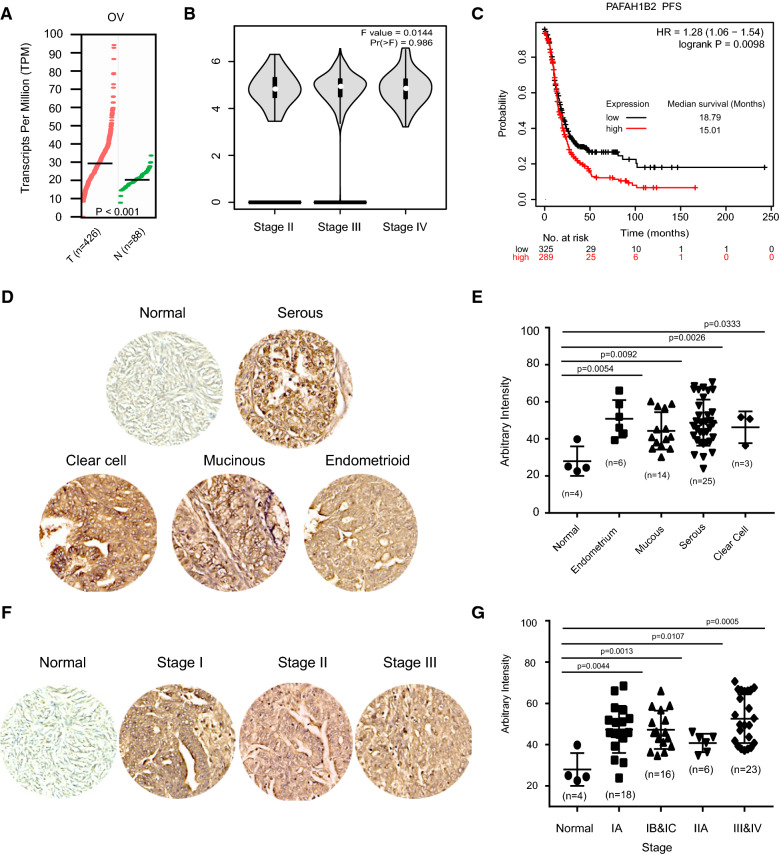
The expression comparison and clinical significance of PAF-AH 1B2 in ovarian cancer. The expression pattern of PAF-AH 1B2 in ovarian tumor and NT (normal tissue) are shown in Wilcoxon signed ranks test (**A**) and stage plot (**B**), including 426 tumor tissues (TCGA) and 88 normal tissues (GTEx). The comparison of PAF-AH 1B2 mRNAexpression level were performed. The log2 (TPM + 1) for log-scale was analyzed for stage plot. PFS (P = 0.0098, **C**) of ovarian cancer patients in was significantly positively associated with the expression of PAF-AH 1B2. *P < 0.05. Scale bars, 100 mm. Representative images are shown the expression pattern of PAF-AH 1B2 in normal tissues, various types (**D**) and stages (**F**) of ovarian tumor. The IHC staining scores were statistic analyzed between normal and tumor tissues (**E** and **G**)

Through the GEPIA Cancer Genomic database, which incorporates a number of published cancer datasets from TCGA [28], PAF-AH IB2 was shown to be overexpressed in 426 ovarian cancer cases compared to normal ovarian tissue (n = 88, Fig. 1A) and was found to be dispersed from stage II to stage IV (Fig. 1B). Patients with higher levels of PAF-AH 1B2 expression had a significantly shorter survival time (PFS, median survival time: 15.01 months, P = 0.0098, Fig. 1C). According to immunohistochemical (IHC) staining in ovarian tumour tissue microarray, PAF-AH IB2 was significantly overexpressed in four subtypes of ovarian tumor tissues (Fig. 1D, E) and at all stage’s cases (Fig. 1F, G).

**Fig. 2:**
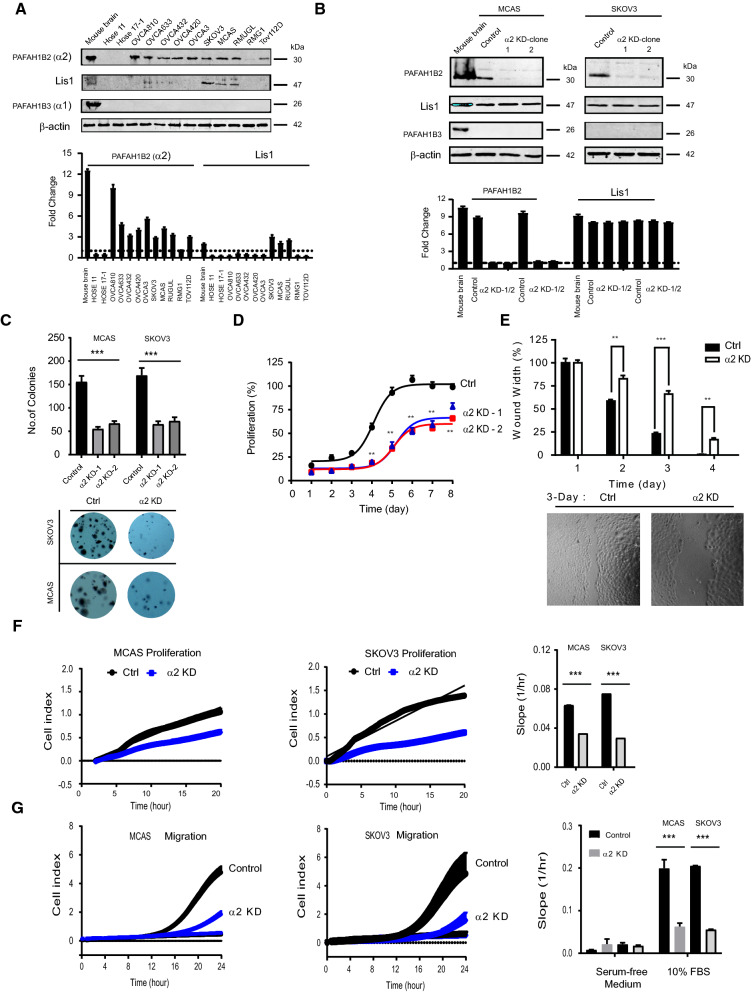
The PAF-AH 1B2 mediated the abnormal proliferation and migration in ovarian cancer cell. Compared the expression of PAF-AH IB2 subunits in ovarian cancer cell lines lysate by Western blot (**A**). Validated the PAF-AH 1B2 stable knockdown effect in the ovarian cancer cells by Western blot (**B**). The comparisons were performed between PAF-AH IB2 and control in the clonogenicity in soft agar (**C**), cell proliferation in 7 days (**D**), wound healing migration (**E**). For the dynamic proliferation test, cells (MCAS, SKOV3, 2.0 × 10^4^ cells/well) were planted by duplicate in the indicated ECM-coated E plates; a non-coated well was used as a negative control (**F**). Cell proliferation curves were monitored through the xCELLigence system (left panel). The rates of cell proliferation over 24 h (slope) were monitored using the RTCA software (right panel). For dynamic migration assay, cells (MCAS, SKOV3, 2.0 × 10^4^ cells/well) were planted by duplicate in the upper chambers of CIM plates, and the lower chambers were contained with 10% FBS (**G**). The migration curves were monitored through the xCELLigence system (left panel) and the migration rates over 24 h (Slope) were processed using the RTCA software (right panel). A representative experimental result was generated from three independent experiments. **P < 0.01 and ***P < 0.001 as compared to control cells expressing a scramble shRNA control, paired *t* test

Furthermore, when compared to human normal ovarian epithelium (HOSE II, HOSE 2282), western blot analysis revealed that PAF-AH IB2 was overexpressed in multiple ovarian cancer cell lines and was partially associated with LIS1 subunits overexpression in MCAS, SKOV3 and RMUGL (Fig. 2A), including MCAS, SKOV3, Tov112D, OVCA3, OVCA420, OVCA432, OVCA633, OVCA810 and RMUGL (Fig. 2A), but a negative signal in RMG1 cell. Interesting, despite a positive signal detected in mouse brain lysate, we were unable to detect the expression signal of the homologue subunit (PAF-AH IB3) in the ovarian cancer cells using western blot. It is thought that neither normal ovarian epithelial cells nor ovarian cancer cells express PAF-AH IB3.

### Knockdown PAF-AH 1B2 impaired the cellular functions of ovarian cancer cell

PAF-AH IB2 wild-type cell lines (Ctrl) and PAF-AH IB2 knockdown cells were successfully transduced with control shRNA or PAF-AH IB2 shRNA constructs using lentivirus, resulting in PAF-AH IB2 wild-type cell lines (Ctrl) and PAF-AH IB2 knockdown cells, respectively (Fig. 2B). In cancer cells (MCAS and SKOV3), knocking down PAF-AH IB2 did not result in homologous subunit (PAF-AH IB3) or other component (Lis1) expression compensatory (Fig. 2B). When compared to the control cell lines, the knockdown cell lines showed a significantly lower ability to form colonies in vitro in soft agar (Fig. 2C, P < 0.001), a significantly slower rate of proliferation (Fig. 2D), and a decrease in cell migratory capacities (Fig. 2E, MCAS). Furthermore, the xCELLigence system was employed to get the dynamic information regarding proliferation and investigate if knocking down ovarian cancer cells (MCAS, SKOV3) affects their proliferation and migratory abilities. PAF-AH 1B2 knockdown cells proliferated at a significantly slower pace than control cells (Fig. 2F, P < 0.001). PAF-AH knockdown 1B2 consistently reduced the ovarian cancer cells’ ability to migrate (Fig. 2G).

### Growth inhibition of non-hydrolysable PAF analogues on ovarian cancer cells

PAF-like ether lipid analogues with non-hydrolyzable sn-2 side chains demonstrated the tumor cell-directed cytotoxicity in vitro [29, 30]. To investigate the efficacy of PAF and its non-hydrolysable analogues in causing tumor cell cytotoxicity, we treated ovarian cancer cell lines with various dosages of PAF and two non-hydrolysable analogues: C-PAF (an N-methylcarbamyl moiety at the sn2 position) and edelfosine (a methyl ether linkage at the sn2 position, Additional file 1: Fig. S1A) and calculated the IC_50_ value in each cancer cell, respectively. PAF exhibited a modest cytotoxicity on ovarian cancer cells (MCAS, TOV112D, RMGUAL, SKOV3 and OVCA3), but had no cytotoxic effect on RMG1 (Additional file 1: Fig. S1B–G). PAF would be digested faster in these cancer cells that expressed higher levels of PAF-AH IB2 (Fig. 2A). C-PAF or edelfosine treatment, on the other hand, demonstrated significant cytotoxicity on these ovarian cancer cells (Additional file 1: Fig. S1B–G).

### Transcriptome analysis discovered key functions and pathways regulated by PAF-AH 1B2 in ovarian cancer cell

**Fig. 3:**
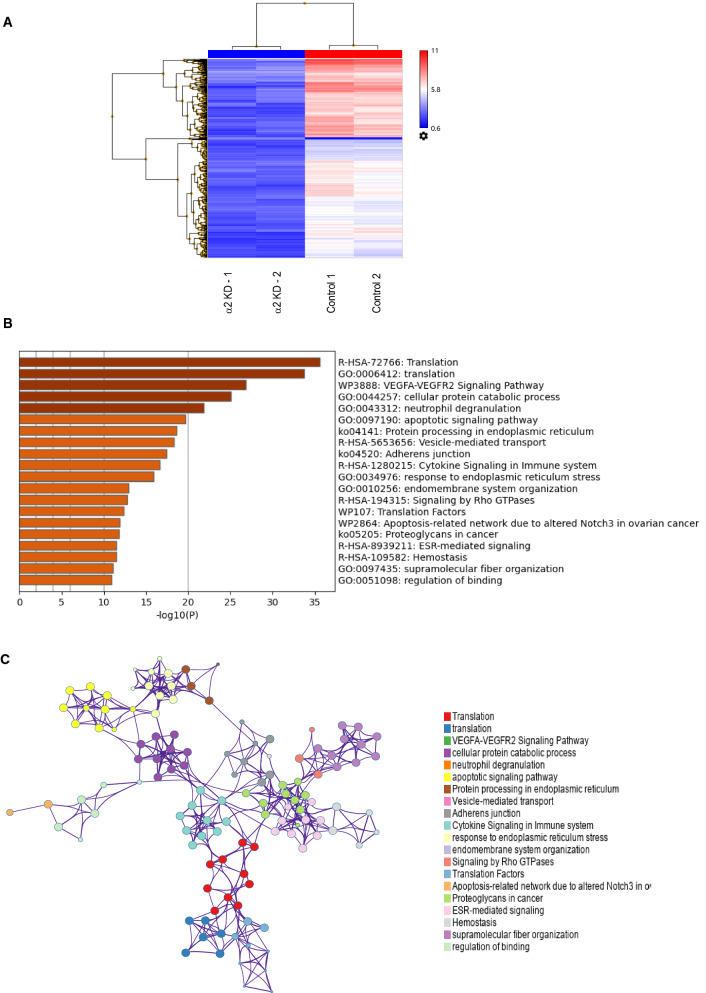
Function and pathway enrichment analysis of the PAF-AH 1B2 knockdown expression profiling in ovarian cancer cells. Heat map demonstrated the significantly changed genes hierarchical cluster analysis of PAF-AH 1B2 knockdown transcription profiles in MCAS ovarian cancer cells. The significant changed genes were screened and identified of PAF-AH 1B2 vs control (**A**). Representative Molecular clusters were enriched. Left panel, heatmap of the top 20 enriched terms (**B**). Representative Molecular Complex Detection (MCODE) network node demonstrated the connection of significantly changed genes regulated by PAF-AH 1B2 knockdown (**C**). Metascape analysis revealed a Network of enriched sets coloured by ID. Threshold value: 0.3 kappa score; similarity score > 0.3. b Heatmap coloured arranged by P-values

According to whole transcriptome profile analysis, the critical functions and key pathways strongly regulated by PAF-AH 1B2 KD in ovarian cancer cells were enriched regulated. 826 genes (up-regulated seven genes; down-regulated 819 genes, Additional file 3: Table S1) were identified as significantly changed and computationally clustered in the **PAF-AH 1B2** KD vs control of MCAS cells (Additional file 3: Table S1, Fig. 3A) after data normalization and significantly analysis filtering (Fold change > 2.0 or < − 2.0, P < 0.001). The significant changed genes were blast through Metascape to enrich the key GO processes and pathways that regulated by the **PAF-AH 1B2** KD, in order to identify the role and regulatory mechanism of **PAF-AH 1B2** in ovarian cancer. The top twenty significantly enriched functional pathways have been summarized (Additional file 4: Table S2, Fig. 3B), including three categories: GO biological process (BP), pathways and Reactome gene sets. Translation (GO:0006412), endomembrane system organization (GO:0010256), response to endoplasmic reticulum stress (GO:0034976), neutrophil degranulation (GO:0043312), cellular protein catabolic process (GO:0044257), regulation of binding (GO:0051098), apoptotic signaling pathway (GO:0097190) and supramolecular fibre organization (GO:0097435) were among the representative enriched GO functions. The top 10 enriched pathways included the VEGFA-VEGFR2 signaling pathway and apoptosis signaling pathway (Additional file 4: Table S2). Furthermore, the interaction pathways revealed the key functional network controlled by PAF-AH 1B2 (Fig. 3C).

According to these results, PAFAH1B2 may play an important regulatory role in in ovarian cancer cells abnormal cell proliferation and adhesion. In the PAF-AH 1B2 KD cancer cells’ enrichment pathways, the apoptotic signaling pathway and VEGFA-VEGFR2 signaling pathway, in particular, were highlighted and chosen for further investigation.

### PAF-AH 1B2 knockdown caused caspases activation  and G2-M cell cycle arrest 

**Fig. 4:**
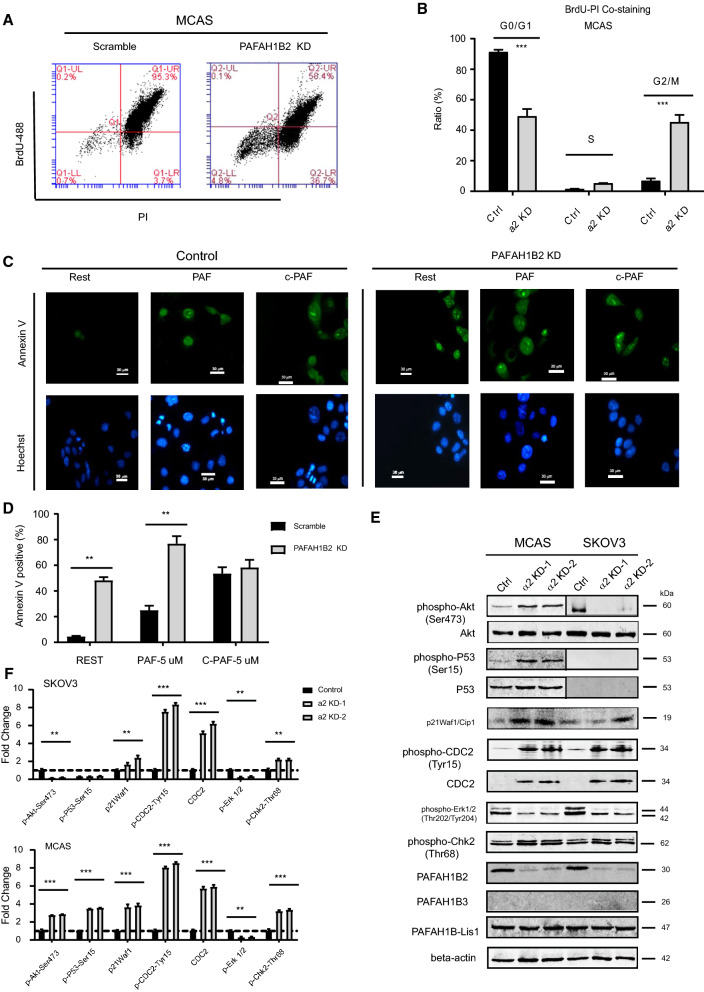
The PAF-AH IB2 KD activated caspases and cell cycle arrest in the ovarian cancer cell. Knockdown PAF-AH 1B2 causes cell cycle arrest of cancer cells identified by BrdU-PI staining analysis (**A**). Representative histograms for cell cycle phase distribution in control and PAF-AH 1B2 KD cells are shown (**B**). Using ANNEXIN V (FITC, green) staining kit by fluorene microscope to evaluate the apoptosis effect induced by PAF-AH 1B2 KD and PAF, C-PAF (**C** and **D**). Data represent as the mean ± S.E.M (n = 10). Western blot detected the signaling molecules change pattern in the PAF-AH 1B2 KD cells that involved in the cell cycle related signaling pathway (**E**) and quantitively analysis (**F**). *P < 0.05; **P < 0.01, and ***P < 0.001 represented as compared with control cells expressing a scramble shRNA control, paired *t* test

Flow cytometric analysis revealed that PAF-AH IB2 knockdown caused cell cycle arrest in ovarian cancer cells (Fig. 4A). The percentages of cell cycle G2/M phase were significantly greater in PAF-AH IB2 knockdown cells than in controls (Fig. 4B, **P < 0.01). Furthermore, PAF-AH IB2 knockdown cells also induced more positive Annexin V signal and were more sensitive to PAF than control cells, although the c-PAF treatment had no effect (Fig. 4C and D). The phosphorylation levels of numerous critical regulatory proteins, including p53-Ser15, Akt-Ser473, CDC2-Tyr15, Chk2-Tyr68, and p21Waf1 and CDC2 that are associated with cell growth and cell cycle arrest regulation [31–34], were significantly increased in PAF-AH 1B2 KD MCAS cells determined by western blot analysis (Fig. 4E), while the phosphorylation of p44/42 MAPK was decreased, which was used as a molecular indicator of tumour cell proliferation and growth. In p53-deficient PAF-AH 1B2 knockdown SKOV3 cells, the Akt-Ser473 phosphorylation level was significantly reduced. Together, the p44/42–Akt–Mdm2-p53 pathway is thought as one of the downstream signalling of PAF-AH IB2, which is responsible for the cell growth inhibition in knockdown ovarian cancer cells.

Furthermore, PAF-AH 1B2 KD significantly increased PAF's growth inhibitory effect and shifted the dose curve in ovarian cancer cells when compared to controls (Additional file 2: Fig. S2A), whereas the cytotoxicity of two non-hydrolyzable analogues, C-PAF and edelfosine (ET-18), on PAF-AH IB2 knockdown and control cells was not significantly different (Additional file 2: Fig. S2B and C). In PAF-AH IB2 knockdown cells, the percentages of positive caspases caspase activated (substrates VAD-FMK) per cells were significantly greater than the controls (Additional file 2: Fig. S2D, **P < 0.01). When PAF-AH IB2 KD cells were treated with C-PAF and edelfosine, the positive signaling of caspase activation staining was not significantly different from control cells. According to western blot analysis, PAF and C-PAF treatment did not induce compensatory expression of PAF-AH 1B3 in PAF-AH 1B2 KD or control cells (Additional file 2: Fig. S2F). In PAF-AH1B2 KD cells, PAF treatment reduced phosphorylation of p44/42 MAPK, whereas C-PAF treatment drastically boosted it. The FAK was upregulated in the PAF-AH 1B2 KD during rest and PAF treatment compared to control cells, but C-PAF treatment showed no effect. These results imply that overexpression of PAH-AH 1B2 in ovarian cancer cells play a critical role in digesting intracellular PAF and reducing PAF-induced caspases activation and apoptosis.

### Over-expression of the catalytic subunits induced human ovarian surface epithelium apoptosis

**Figure. 5:**
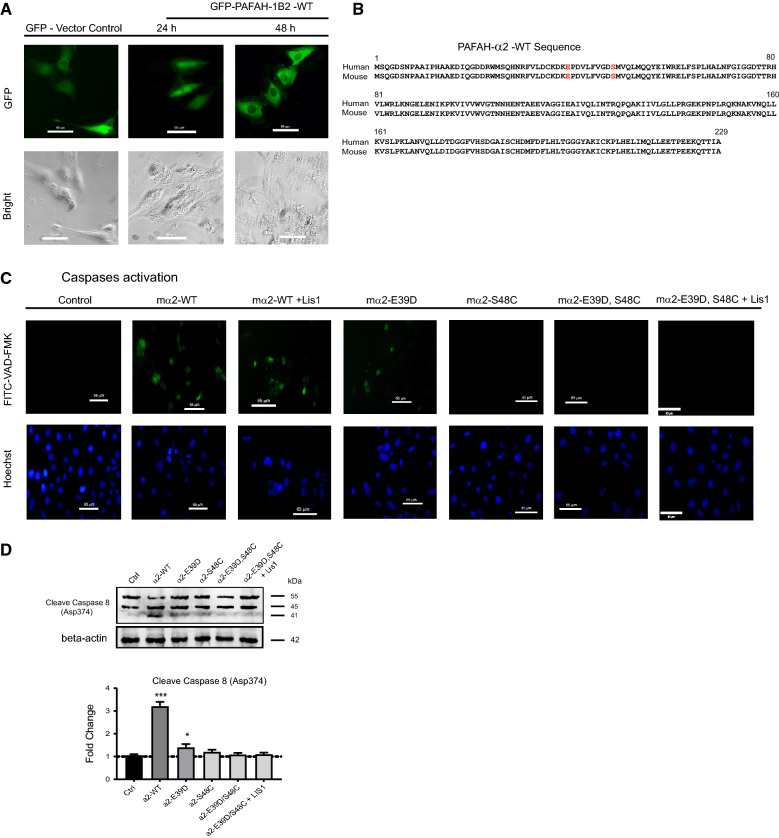
Overexpression of PAF-AH IB2 caused the Caspase-8 activation and normal ovarian epithelium died. Human PAF-AH 1B2 WT transiently over-expression caused the HOSE sick and quickly died after 48-h transfection (**A**). The blank vector linked with GFP was used as control. The protein sequence comparison between human and mouse (**B**). Detection Caspase-8 activation with transfected with mouse PAF-AH 1B2 WT or enzymatic active site mutant (E39D, S48C) in living HOSE (**C**). Western blot validated the Caspase-8 activation and cleavage in the HOSE with mouse PAF-AH 1B2 WT or mutant transfection (**D**). *P < 0.05 and ***P < 0.001 represented as compared with control

Transient over-expression studies on normal HOSE cells were conducted to gain a better understanding of the cellular function of the PAF-AH 1B2. After 48-h transfection, GFP conjugated transient over-expression of human PAF-AH 1B2 WT caused considerable cellular toxicity, resulting in HOSE phenotypic alterations and illness, as well as rapid death (Fig. 5A), whereas pEGFP-C1 vector transfected cells grew properly. Human and mouse PAF-AH 1B2 wild type (WT) protein sequences have been discovered to share 100% identity (Fig. 5B). The Lis1 (human) unit was co-transfected into cells with PAF-AH 1B2 WT or functional mutants (E39D, S48C) (mice), and caspase activation was detected in living cells by FITC-VAD-FMK. The caspases activation was detected in the transfection of mouse PAF-AH 1B2 WT with Lis1 or functional mutants (E39D) (Fig. 5C). Positive caspase activation signals were eliminated when cells were transfected with an enzymatic mutant (S48C) or a dual mutant (E39D, S48C). Furthermore, in HOSE transfected with mouse PAF-AH 1B2 WT and mutants (E39D), endogenous caspase 8 was activated and cleaved, but caspase 8 activation was reduced with mouse PAF-AH 1B2 mutants (S48C) or (E39D, S48C) transfection. Caspase-8 activation was detected by western blot using a cleaved Caspase-8 (Asp374) antibody, which specifically detects endogenous cleavage at aspartic acid 374 (Fig. 5D). After Caspase-8 is activated, downstream effector caspases, such as caspase-1, -3, -6, and -7, are activated. Caspase-3 causes DNA fragmentation and cell shrinkage, which are morphological hallmarks of apoptosis [35]. As a result of PAF-AH 1B2 overexpression, Caspase-8 was activated and caused HOSE death.

### Knockdown PAF-AH 1B2 caused the aberrant-activation of multiple tyrosine kinases signalling pathways down-regulated in ovarian cancer cell

**Fig. 6:**
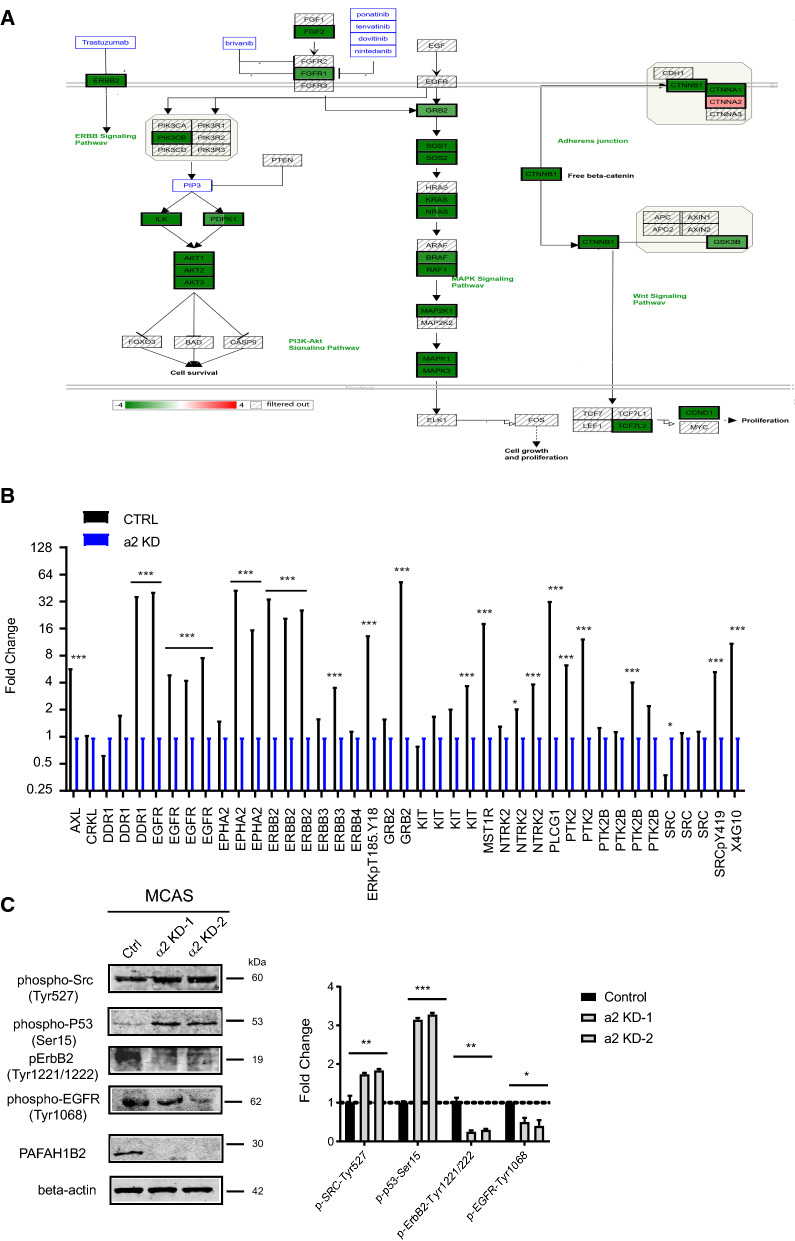
PAF-AH IB2 Knockdown decreased the growth factor kinase pathway in the ovarian cancer cell. The significantly changed genes in PAF-AH 1B2 KD MCAS cells were enriched in the growth factor kinase signalling pathway in endometrial cancer (**A**). Color indicates significantly up-regulated signals and down-ward (green) ones indicate down-regulated expression levels of the genes. Luminex immune-assay screen identified activated tyrosine kinases in PAF-AH 1B2 KD cancer cell lines (**B**). Western blot validated the signaling molecules change pattern in the PAF-AH 1B2 KD cells that involved in the epithermal growth factor kinase related signaling pathway (**C**). *P < 0.05; **P < 0.01, and ***P < 0.001 represented as compared with control

The VEGFA-VEGFR2 signaling pathway was highlighted in the enrichment pathways of PAF-AH 1B2 KD cancer cells, and the related signaling pathway network and significantly changed genes were visualized by exploring through the Wiki Cancer network (Fig. 6A), which contained several tyrosine kinases and downstream signalling pathways, such as MAPK and PI3K/AKT, to regulate cell proliferation and growth. Upward thermometers are red and indicate up-regulated signals, whereas descending thermometers are green and indicate down-regulated gene expression levels. The major genes that were down-regulated included FGFR1, GRB2, ERBB2, and MAPK1.

The aberrant tyrosine kinase activity was investigated to see how PAF-AH 1B2 KD impacted on the profiling of tyrosine kinase activation. The phosphorylation status of EGFR, ERBB2, GRB2, and SRC were tested using a Luminex assay, and found to be significantly decreased in the PAF-AH 1B2 KD cells (Fig. 6B). The results support previous research findings that tyrosine phosphorylation mediates signaling proteins interactions with EGFR. Furthermore, western blot analysis demonstrated that the phosphorating levels of ERBB2 (Tyr1221/1222) and EGFR (Tyr1068) were decreased in PAF-AH 1B2 KD cells, whereas SRC was increased (Fig. 6C). PA-FAH 1B2, which is abnormally overexpressed in ovarian cancer and promotes proliferation, is hypothesized to play a role in oncogenic multiple tyrosine kinases-mediated cellular transformation.

## Discussion

In this study, PAF-AH 1B2 was identified to be a novel potential biomarker that is significant in driving aggressive and tumorigenic features of ovarian cancer through exploring TCGA database. PAF-AH 1B2 degrades PAF intracellularly to maintain key reproduction function in ovarian [12]. The overexpression pattern of PAF-AH 1B2 was confirmed in clinic ovarian cancer samples using immunohistochemical (IHC) staining with ovarian cancer tissue microarray (Fig. 1C and D). In ovarian cancer samples that identified by IHC, the protein expression level of PAF-AH 1B2 was significantly higher than in normal ovarian tissue. However, the expression of a1 subunit was not found in the tumor tissues (data not show). When compared to normal human ovarian surface epithelium, the abnormally up-regulated pattern of α2 was confirmed by western blot in the majority of ovarian cancer cell lines (Fig. 2A). Our findings suggest that the overexpression of PAF-AH 1B2 in ovarian cancer and normal ovarian physiologic function is mediated by an unmask dysregulation network. Platelet activating factor acetyl hydrolase 1B2 and 1B3 (PAF-AH 1B2 and PAF-AH 1B3) were previously found to be overexpressed or their activities abnormally upregulated in a variety of cancers [3, 15, 18], worked as a metabolic driver in the pathogenicity of breast cancer [15], correlated with pancreatic ductal adenocarcinoma patients’ poor survival, affected proliferation and apoptosis in Osteosarcoma [18], and mediated the lipid and mediated by several oncogenes, such as HIF1α mediated PAF-AH 1B2 overexpression in PDAC [16]. PAF-AH 1B2 overexpression was identified in numerous ovarian cancer cells in our investigation, not PAF-AH 1B3. Previous PAF-AH 1B2-related investigations have identified aberrant regulatory effects on proliferation and survival in a variety of cancer types, and our findings were consistent with them. It’s possible that PAF-AH 1B2 /B3 has a different dominating role in different tumor types.

Lipid metabolism, which provides energy and nutrients as well as signalling for tumor survival, growth, and metastasis, has specific implications in signalling of tumour survival, growth, and metastasis [36]. Many lipid metabolites, such as lysophosphatidic acid (LPA) and platelet-activating factor (PAF), are bioactive lipids that work as the second messengers to initiate signalling cascades for ovarian cancer and metastasis [37, 38]. Platelet-activating factor (PAF) is a phospholipid that involved in the inflammation, migration and cell invasion [39, 40]. PAF synthesis, transport, and enzymatic degradation are all tightly regulated and linked to a wide range of physiologic processes [40]. PAF intracellular degradation is predominantly handled by the PAF-AH 1B2 enzyme. PAF receptor-independent pathway can also absorb extracellular PAF, resulting in caspase-3-dependent apoptosis. PAF-AH 1B2 expression and the duration of pathological cytosolic PAF accumulation could be influenced by the apoptogenic concentration of extracellular PAF [41]. In our study, even at a relatively high concentration (5 µM), PAF had only a minor growth inhibitory effect on several ovarian cancer cells, whereas the cytotoxic effects of c-PAF and edelfosine, which are PAF-like non-hydrolysable ether lipid analogues that selectively kill tumor cells while sparing normal cells, were quite strong at the same concentration [42]. It’s been proposed that ovarian cancer cell lines have strong intracellular enzymic activity to counteract the PAF’s cytotoxic effect. Our findings revealed a counter-balance network of PAF-AH 1B2 and PAF in ovarian cancer cells, advancing the understand of PAF-AH 1B2’s dis-regulatory role in oncogenesis.

The physiologic function deficit is caused by abnormal expression of PAF-AH 1B2. In mice, knockout α2 resulted in infertile and impaired spermatogenesis, as well as a decrease in Lis1 protein expression [43, 44]. PAF-AH 1B2 knockdown cancer cells were considerable less able to proliferate, migrate (Fig. 2D), and forming colonies in vitro in soft agar (Fig. 2C) was significantly reduced. Because the expression pattern of the other subunits, particularly α1 (PAF-AH 1B3), is unaffected, abnormal enzymatic activity of PAF-AH 1B2 appears to play a dominant role in ovarian cancer. Furthermore, knocking out PAF-AH 1B2 significantly increased PAF’s cytotoxic effect on cancer cells (Additional file 2: Fig. S2A). It corresponds to the PAF-AH 1B2’s intracellular counterbalancing role. This result matched the previous results that PAF-AH I α2 worked as a potent anti-apoptotic protein and inhibited PAF-mediated cell death [41]. According to these results, PAF-AH 1B2 has the potential to be a synergistic chemotherapeutic target for ovarian cancer.

Furthermore, knockdown PAF-AH 1B2 caused caspases activation and cancer cells cycle arrest, apoptosis, as well as significant rise the phosphorylation levels of related regulatory proteins p53-Ser15, Akt-Ser473, CDC2-Tyr15, Chk2-Tyr68, and the protein level of p21Waf1 and CDC2 (Fig. 4E), all of which have been associated with relate regulation [32, 33]. According to a previous study, LPA can induce p21^Waf1^ expression and mediate cytostatic response in cancer cells [45], which is consistent with our findings. Combined previously results, it hints that there is a novel regulation network that knockdown of PAF-AH IB2 might cause a feedback loop that leads to increased biosynthesis of MAGE and LPA. LPA exhibits pleiotropic biological functions, depending on which G protein-coupled receptors (GPCR) it interacts with [46, 47]. Our results will enhance the understand the role of intracellular lipid metabolites including MAGE, LPA and PAF in cancer progress, as well as the connection between GPCR signaling and ovarian cancer.

It is well established that α2 has a stronger affinity for LIS1 than does α1 subunit [44]. PAF-AH 1B2 could form homodimer, and the position of Glu39 is critical for binding with Lis1 and Ser47 is key site of the catalytic centre [19, 44]. Both the catalytically active and inactive mutant forms of PAF-AH 1B2 (E39D, S48C, E39D/S48C), with or without Lis1, were transiently overexpressed in HOSE cells to investigate the mechanism of PAF-AH 1B2 abnormal expression and determine whether this enzyme was sufficient to confer oncogenic properties.

Endogenous Caspase-8 was cleaved and HOSE died soon after overexpression of the wild-type form of PAF-AH 1B2 activated caspases (Fig. 5A, C and D). These favourable activation and phenotypic changes were completely reversed in the catalytically inactive mutant (S48C, E39D/S48C). Since PAF-AH 1B2 has been shown to interact with PAF-AH 1B1, Lis1, our results also indicated that even co-overexpression Lis1 with PAF-AH 1B2 WT may be not sufficient to induce the abnormally proliferation in the HOSE, hinting missed a link between over-expression of the PAF-AH 1B2 and ovarian cancer pathogenesis. It is strongly suggested that some newly un-discovered materials, when combined with PAF-AH 1B2 overexpression, can transform normal HOSE into cancerous characteristics. Furthermore, the phosphorylation levels of EGFR, ERBB2, GRB2, and SRC were significantly reduced in the PAF-AH 1B2 KD cancer cells as determined by the Luminex assay and western blot data (Fig. 6B). These results support previous findings that PAF-PAFR signaling pathway could synergistically be activated with tyrosine kinase -VEGFR pathway to modulate the abnormal proliferation in ovarian cancer [48, 49]. PAF-AH 1B2, which is abnormally overexpressed in ovarian cancer and promotes proliferation, is thought to act as the essential signaling mediator of oncogenic tyrosine kinases signalling pathways mediated cellular transformation.

## Conclusions

In conclusion, our results shed new light on the role of the PAF-AH IB2 and regulated pathways in ovarian pathogenesis, leading to the identification of new marker and signalling for ovarian cancer, as well as new potential preventive and therapeutic strategies that target the enzyme. Our findings, along with those of others, show a novel interaction network of lipid metabolic PAF-AH 1B2 and other enzymes in ovarian cancer. In order to further understand how PAF-AH 1B2 regulates the metabolic and signalling pathways in ovarian pathogenesis. future study will focus on elucidating signal transduction from ether lipid messengers to downstream pathways. Another line of research will focus on the regulation mechanism of PAF-AH 1B2 abnormal expression in ovarian cancer.

## Supplementary Information

**Additional file 1: Figure S1.** Characterized the growth inhibitory effect of PAF and non- PAF analogue on the ovarian cancer cells. The structure analysis between PAF and non-PAF analogue ester lipid drugs (A). The growth inhibitory evaluation among multiple ovarian cancer cells, including MCAS (B), TOV112D (C), RMUGL (D), OVCA3 (E), SKOV3(F) and RMG1(G).

**Additional file 2: Figure S2.** The comparison growth inhibited effect of PAF and non-hydrolysable PAF analogues on PAF-AH 1B2 Knockdown ovarian cancer cell lines. The comparison cytotoxic effect of PAF (A) and analogue c-PAF (B) and Edelfosine (C) on PAF-AH 1B2 Knockdown and control ovarian cancer cells were tested by MTT-based assay. Typical representation images of cells that rest condition and treated with PAF and non-hydrolysable PAF analogues stained with FITC-VADfmk (D). The cells were counterstained with the nuclear dye Hoechst33342. Western blot detected the signaling molecules change pattern in the PAF-AH 1B2 KD cells that involved in the cell cycle related signaling pathway (E).

**Additional file 3: Table S1.** The significant change genes between PAF-AH 1B2 KD and control cells.

**Additional file 4: Table S2.** Top 20 representative enriched clusters by go biological processes.
