# Supplementary figures and images for: Platelet-activating factor acetyl hydrolase IB2 dysregulated cell proliferation in ovarian cancer
Source: Cancer Cell Int. 2021 Dec 20;21:697. doi: 10.1186/s12935-021-02406-9 (PMC8690939; doi:10.1186/s12935-021-02406-9)

A

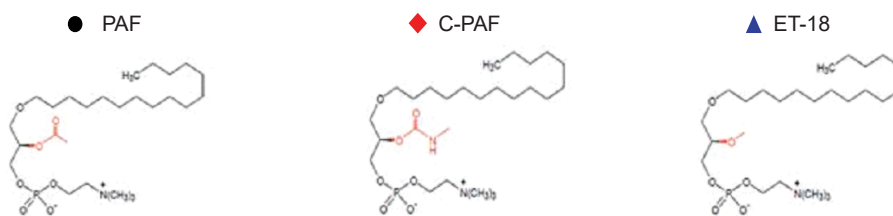

B

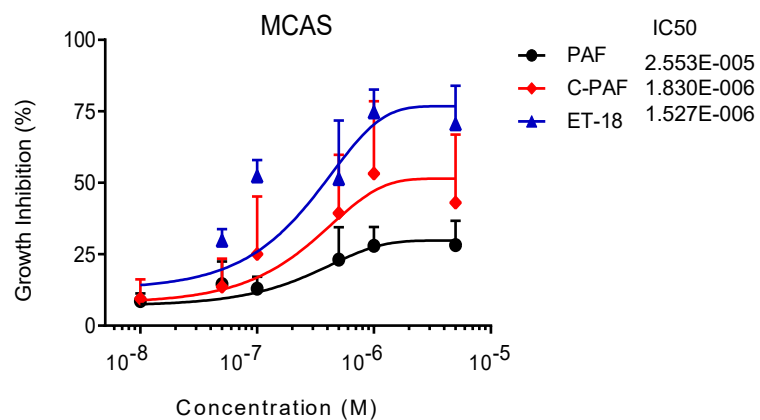

C

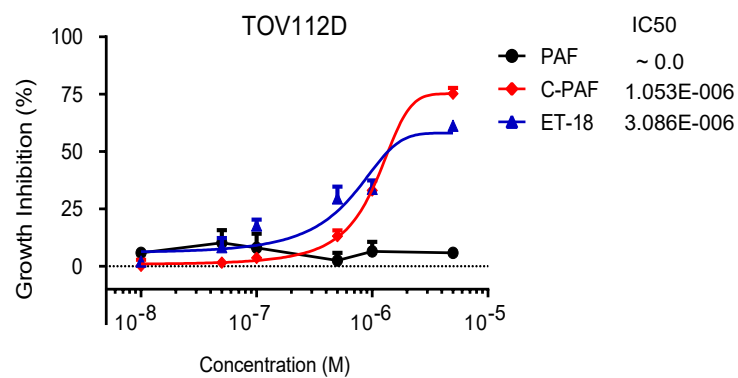

D

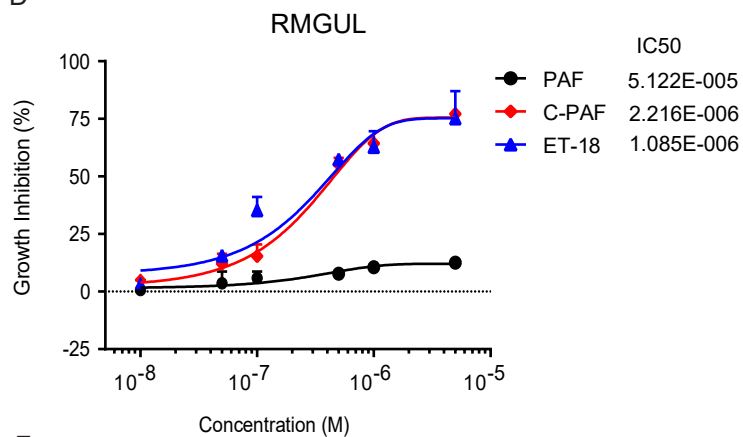

E

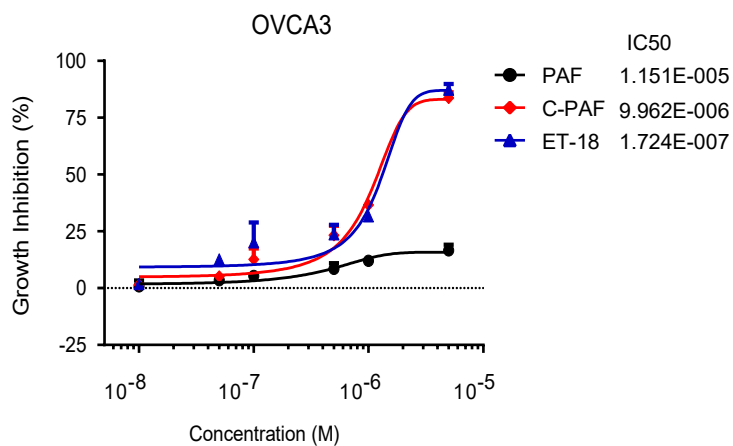

F

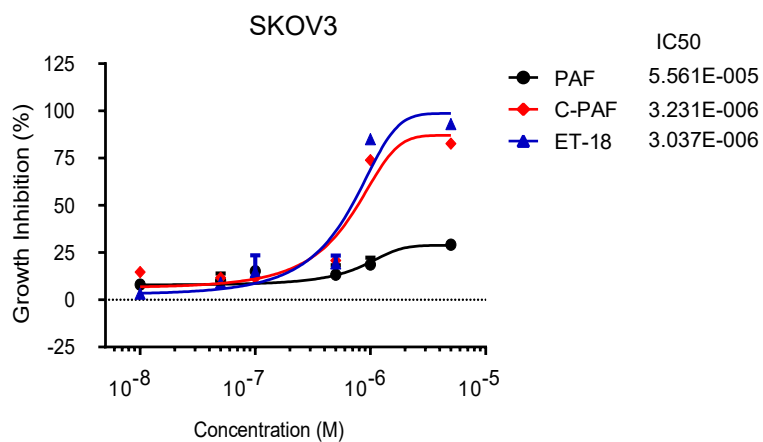

G

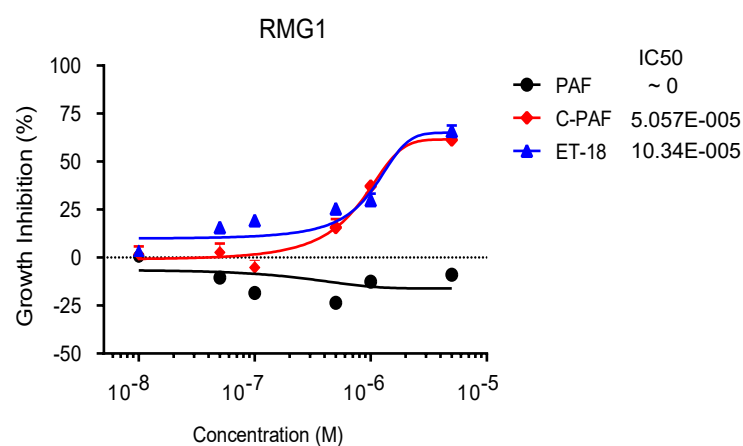

Supplement: Supplementary file 1 — Additional file 1: Figure S1. Characterized the growth inhibitory effect of PAF and non- PAF analogue on the ovarian cancer cells. The structure analysis between PAF and non-PAF analogue ester lipid drugs (A). The growth inhibitory evaluation among multiple ovarian cancer cells, including MCAS (B), TOV112D (C), RMUGL (D), OVCA3 (E), SKOV3(F) and RMG1(G). [file 12935_2021_2406_MOESM1_ESM.pdf]

A

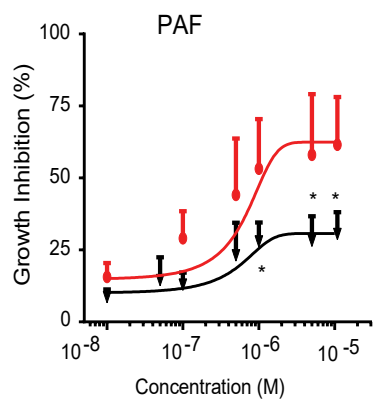

B

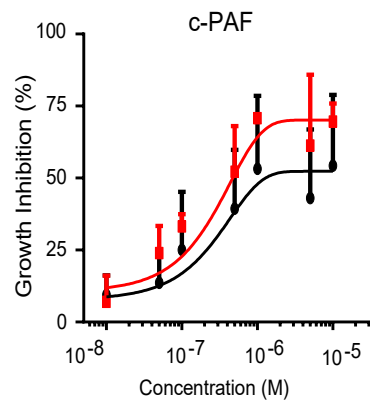

C

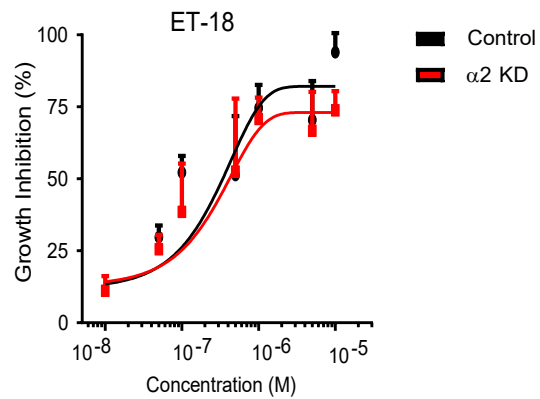

D

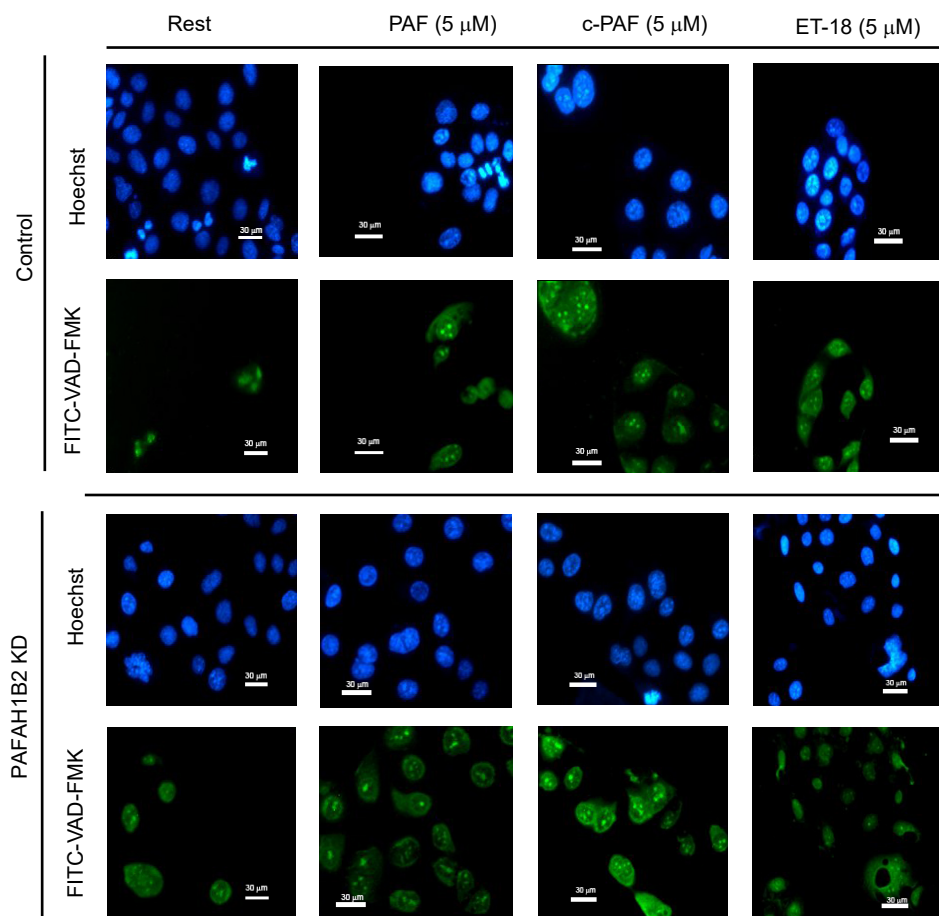

E

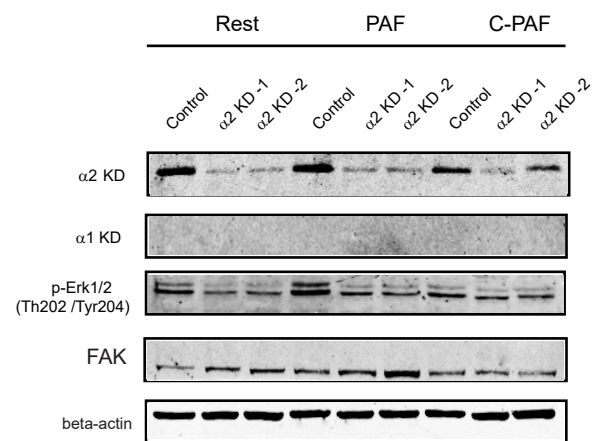

Supplement: Supplementary file 2 — Additional file 2: Figure S2. The comparison growth inhibited effect of PAF and non-hydrolysable PAF analogues on PAF-AH 1B2 Knockdown ovarian cancer cell lines. The comparison cytotoxic effect of PAF (A) and analogue c-PAF (B) and Edelfosine (C) on PAF-AH 1B2 Knockdown and control ovarian cancer cells were tested by MTT-based assay. Typical representation images of cells that rest condition and treated with PAF and non-hydrolysable PAF analogues stained with FITC-VADfmk (D). The cells were counterstained with the nuclear dye Hoechst33342. Western blot detected the signaling molecules change pattern in the PAF-AH 1B2 KD cells that involved in the cell cycle related signaling pathway (E). [file 12935_2021_2406_MOESM2_ESM.pdf]
