# Supplementary material for: Platelet-activating factor acetyl hydrolase IB2 dysregulated cell proliferation in ovarian cancer
Source: Cancer Cell Int. 2021 Dec 20;21:697. doi: 10.1186/s12935-021-02406-9 (PMC8690939; doi:10.1186/s12935-021-02406-9)
Supplement: Supplementary file 3 — Additional file 3: Table S1. The significant change genes between PAF-AH 1B2 KD and control cells. [file 12935_2021_2406_MOESM3_ESM.pdf]

Table S1 The signficant change genes between PAFAH1B2 KD and control cells.

Gene Symbol	Description	Fold Change	P-val	FDR P-val
AARS	alanyl-tRNA synthetase	-18.91	0.0001	0.0237
AASDHPPT	aminoadipate-semialdehyde dehydrogenase-phosphopantetheinyl transferase	-7.02	0.0005	0.0293
ABHD13	abhydrolase domain containing 13	-6.7	0.0010	0.0351
ACER3	alkaline ceramidase 3	-15.49	0.0006	0.0306
ACTB	actin, beta	-67.67	0.0003	0.0261
ACTN1	actinin, alpha 1	-40.9	0.0001	0.0237
ACTN4	actinin, alpha 4	-57.61	0.0006	0.0306
ADIRF	adipogenesis regulatory factor; ankyrin repeat and GTPase domain Arf GTPase activating protein 11	-4.35	0.0010	0.0351
ADRM1	adhesion regulating molecule 1	-14.3	0.0004	0.0269
AES	amino-terminal enhancer of split	-4.84	0.0008	0.0332
AFF4	AF4/FMR2 family, member 4	-9.46	0.0003	0.0252
AGFG1	ArfGAP with FG repeats 1	-18.81	0.0002	0.0238
AHNAK2	AHNAK nucleoprotein 2	-13.97	0.0002	0.0238
AKT1	v-akt murine thymoma viral oncogene homolog 1	-19.42	0.0003	0.0252
AMFR	autocrine motility factor receptor, E3 ubiquitin protein ligase	-13.1	0.0003	0.0262
AMZ2	archaelysin family metallopeptidase 2	-25.96	0.0002	0.0243
ANKIB1	ankyrin repeat and IBR domain containing 1	-13.9	0.0002	0.0243
ANKRD10	ankyrin repeat domain 10; ANKRD10 intronic transcript 1	-10.13	0.0002	0.0243
ANO6	anoctamin 6	-15.05	0.0008	0.0331
ANP32B	acidic nuclear phosphoprotein 32 family member B	-6.69	0.0004	0.0271
ANXA2	annexin A2	-11.68	0.0008	0.0331
ANXA2P2	annexin A2 pseudogene 2	-38.78	1.93E-05	0.0237
ANXA3	annexin A3	-21.54	0.0007	0.0312
ANXA4	annexin A4	-8.79	0.0008	0.0331
ANXA5	annexin A5	-12.2	0.0008	0.0331
ANXA7	annexin A7	-4.84	0.0010	0.0351
AP3D1	adaptor-related protein complex 3, delta 1 subunit	-6.29	0.0006	0.0309
APEX1	APEX nuclease (multifunctional DNA repair enzyme) 1	-17.3	0.0002	0.0238
API5	apoptosis inhibitor 5	-38.01	8.46E-05	0.0237
APLP2	amyloid beta (A4) precursor-like protein 2	-23.54	0.0007	0.0320
APP	amyloid beta (A4) precursor protein	-13.12	0.0002	0.0238
ARCN1	archain 1	-17.04	0.0005	0.0282
ARF1	ADP-ribosylation factor 1	-11.32	0.0007	0.0319
ARF3	ADP-ribosylation factor 3	-29.67	2.96E-05	0.0237

ARF4	ADP-ribosylation factor 4	-24.15	0.0001	0.0237
ARF5	ADP-ribosylation factor 5; fascin actin-bundling protein 3, testicular	-9.04	0.0008	0.0331
ARHGAP1	Rho GTPase activating protein 1	-10.9	0.0005	0.0294
ARHGEF12	Rho guanine nucleotide exchange factor (GEF) 12	-11.2	0.0002	0.0243
ARIH1	ariadne RBR E3 ubiquitin protein ligase 1	-17.78	0.0001	0.0237
ARL1	ADP-ribosylation factor like GTPase 1	-6.36	0.0004	0.0269
ARL2BP	ADP-ribosylation factor like GTPase 2 binding protein	-13.06	0.0004	0.0271
ARL6IP5	ADP-ribosylation factor like GTPase 6 interacting protein 5	-28.59	5.50E-05	0.0237
ARPC1B	actin related protein 2/3 complex subunit 1B; actin related protein 2/3 complex subunit 1A	-9.36	0.0002	0.0238
ARPC2	actin related protein 2/3 complex subunit 2	-90.21	1.69E-05	0.0237
ARPC3	actin related protein 2/3 complex subunit 3	-20.16	8.08E-05	0.0237
ASAH1	N-acylsphingosine amidohydrolase (acid ceramidase) 1	-14.74	0.0005	0.0282
ASNA1	arsA arsenite transporter, ATP-binding, homolog 1 (bacterial)	-11.98	0.0003	0.0252
ATF1	activating transcription factor 1	-10.25	0.0008	0.0326
ATF2	activating transcription factor 2	-10.1	0.0003	0.0252
ATF6	activating transcription factor 6	-14.06	0.0001	0.0237
ATMIN	ATM interactor	-18.88	0.0006	0.0305
ATP1A1	ATPase, Na+/K+ transporting, alpha 1 polypeptide	-26.3	7.33E-05	0.0237
ATP2A2	ATPase, Ca++ transporting, cardiac muscle, slow twitch 2	-13.59	0.0010	0.0351
ATP5B	ATP synthase, H+ transporting, mitochondrial F1 complex, beta polypeptide	-48.42	0.0010	0.0351
ATP5G1	ATP synthase, H+ transporting, mitochondrial Fo complex subunit C1 (subunit 9)	-29.56	0.0010	0.0351
ATP6V0C	ATPase, H+ transporting, lysosomal 16kDa, V0 subunit c	-8.78	0.0009	0.0343
ATP6V0E1	ATPase, H+ transporting, lysosomal 9kDa, V0 subunit e1	-10.29	0.0004	0.0271
ATRAID	all-trans retinoic acid-induced differentiation factor	-15.77	0.0001	0.0237
ATXN10	ataxin 10	-21.03	0.0001	0.0237
AUP1	ancient ubiquitous protein 1	-12.14	0.0001	0.0237
BASP1	brain abundant, membrane attached signal protein 1	-9.85	0.0002	0.0243
BHLHE40	basic helix-loop-helix family, member e40	-14.2	0.0005	0.0282
BNIP3L	BCL2/adenovirus E1B 19kDa interacting protein 3-like	-7.59	0.0003	0.0261
C11orf58	chromosome 11 open reading frame 58	-9.89	0.0004	0.0265
C12orf57	chromosome 12 open reading frame 57	-8.01	0.0007	0.0312
AP3S2	C15orf38-AP3S2 readthrough; adaptor-related protein complex 3, sigma 2 subunit	-9.72	0.0007	0.0313
C19orf53	chromosome 19 open reading frame 53	-23.52	3.71E-05	0.0237
C1orf174	chromosome 1 open reading frame 174	-9.45	0.0005	0.0277
TGIF2	chromosome 20 open reading frame 24; TGIF2-C20orf24 readthrough	-10.4	0.0002	0.0243

DDX46	chromosome 5 open reading frame 24; DEAD (Asp-Glu-Ala-Asp) box polypeptide 46	-11.01	0.0007	0.0312
CAB39	calcium binding protein 39	-8.94	0.0007	0.0317
CALB1	calbindin 1	-22.11	0.0004	0.0270
CAMK2N1	calcium/calmodulin-dependent protein kinase II inhibitor 1	-18.68	0.0006	0.0306
CANX	calnexin	-89.15	1.99E-05	0.0237
CAP1	CAP, adenylate cyclase-associated protein 1 (yeast)	-15.7	0.0002	0.0238
CAPN2	calpain 2, (m/II) large subunit	-14.79	0.0002	0.0238
CAPRIN1	cell cycle associated protein 1	-21.76	0.0007	0.0311
CAPZB	capping protein (actin filament) muscle Z-line, beta	-21.38	0.0001	0.0237
CAST	calpastatin	-12.06	0.0005	0.0293
CAV1	caveolin 1	-87.86	0.0002	0.0243
CAV2	caveolin 2; caveolin 1	-24.32	0.0003	0.0252
CBFB	core-binding factor, beta subunit	-9.17	0.0002	0.0238
CBLL1	Cbl proto-oncogene-like 1, E3 ubiquitin protein ligase	-10.46	0.0004	0.0271
CBX5	chromobox homolog 5	-50.2	0.0002	0.0238
CCAR1	cell division cycle and apoptosis regulator 1	-9.12	0.0008	0.0328
CCNC	cyclin C	-11.59	0.0002	0.0243
CCND1	cyclin D1	-7.9	0.0010	0.0351
CCNI	cyclin I	-19.52	0.0002	0.0243
CCNK	cyclin K	-15.18	0.0002	0.0245
CCNT2	cyclin T2	-5.4	0.0006	0.0306
CCT7	chaperonin containing TCP1, subunit 7 (eta)	-18.04	0.0004	0.0271
CCZ1	CCZ1 homolog, vacuolar protein trafficking and biogenesis associated; CCZ1 homolog B, vacuolar protein trafficking and biogenesis associated	-5.29	0.0006	0.0306
CD164	CD164 molecule, sialomucin	-21.76	0.0008	0.0331
CD44	CD44 molecule (Indian blood group)	-12.47	0.0001	0.0237
CD55	CD55 molecule, decay accelerating factor for complement (Cromer blood	-16.73	0.0004	0.0269
CD59	CD59 molecule, complement regulatory protein	-7.65	0.0007	0.0319
CD81	CD81 molecule	-26.24	0.0002	0.0243
CDC123	cell division cycle 123	-8.83	0.0004	0.0265
CDC25B	cell division cycle 25B	-10.93	0.0006	0.0302
CDC42BPB	CDC42 binding protein kinase beta (DMPK-like)	-14.61	0.0007	0.0317
CDC42SE2	CDC42 small effector 2	-10.23	0.0002	0.0243
CDH13	cadherin 13	-23.27	0.0002	0.0238
CDK12	cyclin-dependent kinase 12	-7.75	0.0009	0.0336
CDK17	cyclin-dependent kinase 17	-8.05	0.0003	0.0252
CDK4	cyclin-dependent kinase 4	-34.65	4.86E-05	0.0237

CDS2	CDP-diacylglycerol synthase 2	-12.51	0.0002	0.0238
CDV3	CDV3 homolog (mouse)	-7.81	0.0005	0.0293
CEP95	centrosomal protein 95kDa	-5.84	0.0008	0.0332
CEPT1	choline/ethanolamine phosphotransferase 1	-9.18	0.0005	0.0294
CETN2	centrin 2	-6.05	0.0008	0.0331
CFAP97	cilia and flagella associated protein 97	-5.92	0.0008	0.0331
CGGBP1	CGG triplet repeat binding protein 1	-8.55	0.0006	0.0306
CHIC2	cysteine rich hydrophobic domain 2	-20.32	0.0008	0.0333
CHMP2A	charged multivesicular body protein 2A	-20.74	0.0002	0.0243
CHP1	calcineurin-like EF-hand protein 1	-25.59	8.69E-05	0.0237
CHPT1	choline phosphotransferase 1	-10.95	0.0005	0.0293
CHST11	carbohydrate (chondroitin 4) sulfotransferase 11	-5.51	0.0009	0.0345
CIAO1	cytosolic iron-sulfur assembly component 1	-15.18	0.0002	0.0243
CKS2	CDC28 protein kinase regulatory subunit 2	-21.66	0.0001	0.0237
CLIC1	chloride intracellular channel 1	-12.05	0.0003	0.0259
CLIC1	chloride intracellular channel 1	-9.65	0.0005	0.0282
CLIC1	chloride intracellular channel 1	-10.81	0.0008	0.0331
CLINT1	clathrin interactor 1	-12.35	0.0006	0.0309
CNIH4	cornichon family AMPA receptor auxiliary protein 4	-7.31	0.0004	0.0271
CNOT1	CCR4-NOT transcription complex subunit 1	-14.98	0.0009	0.0346
CNOT11	CCR4-NOT transcription complex subunit 11	-9.74	0.0007	0.0321
BMI1	COMMD3-BMI1 readthrough; BMI1 proto-oncogene, polycomb ring finger	-9.32	0.0003	0.0252
COPA	coatomer protein complex subunit alpha	-17.2	0.0001	0.0237
COPG1	coatomer protein complex subunit gamma 1	-6.93	0.0009	0.0339
COPS3	COP9 signalosome subunit 3	-13.26	0.0007	0.0320
COPZ1	coatomer protein complex subunit zeta 1	-27.22	0.0004	0.0265
COQ5	coenzyme Q5, methyltransferase	-5.56	0.0005	0.0294
CORO1C	coronin, actin binding protein, 1C	-14.92	0.0009	0.0339
COX5B	cytochrome c oxidase subunit Vb	-19.35	0.0009	0.0341
CREB1	cAMP responsive element binding protein 1	-11.01	0.0003	0.0256
CRHR1	corticotropin releasing hormone receptor 1	-15.07	0.0004	0.0269
CSDE1	cold shock domain containing E1, RNA binding; neuroblastoma RAS viral (v-ras) oncogene homolog	-40.55	0.0003	0.0252
NRAS	cold shock domain containing E1, RNA binding; neuroblastoma RAS viral (v-ras) oncogene homolog	-31.09	0.0006	0.0309
CSF1	colony stimulating factor 1 (macrophage)	-9.45	0.0004	0.0271
CSNK2A1	casein kinase 2, alpha 1 polypeptide	-21.55	5.70E-05	0.0237
CSNK2A2	casein kinase 2, alpha prime polypeptide	-27.34	5.89E-05	0.0237

CSNK2B	casein kinase 2, beta polypeptide; lymphocyte antigen 6 complex, locus G5B	-41.41	0.0010	0.0351
CSRP1	cysteine and glycine-rich protein 1	-22.55	0.0001	0.0237
CSTB	cystatin B (stefin B)	-13.65	0.0003	0.0252
CTDSPL2	CTD small phosphatase like 2	-19.29	0.0004	0.0271
CTNNA1	catenin (cadherin-associated protein), alpha 1	-13.05	0.0003	0.0256
CTNNB1	catenin (cadherin-associated protein), beta 1	-26.52	0.0001	0.0237
CTNND1	catenin (cadherin-associated protein), delta 1; TMX2-CTNND1 readthrough (NMD candidate)	-17.17	0.0004	0.0271
CTSB	cathepsin B	-15.48	0.0001	0.0238
CTSD	cathepsin D	-34.77	0.0001	0.0237
CUL1	cullin 1	-8.41	0.0005	0.0287
CUL3	cullin 3	-8.46	0.0006	0.0306
CXXC5	CXXC finger protein 5	-4.47	0.0009	0.0343
CYB5B	cytochrome b5 type B (outer mitochondrial membrane)	-12.6	0.0001	0.0237
DCAF12	DDB1 and CUL4 associated factor 12	-9.44	0.0008	0.0331
DCTN4	dynactin 4 (p62)	-8.76	0.0005	0.0282
DCUN1D4	DCN1, defective in cullin neddylation 1, domain containing 4	-7.08	0.0008	0.0331
DCXR	dicarbonyl/L-xylulose reductase	-15.49	0.0007	0.0318
DDOST	dolichyl-diphosphooligosaccharide--protein glycosyltransferase subunit (non-catalytic); PINK1 antisense RNA	-8.03	0.0005	0.0294
DDX17	DEAD (Asp-Glu-Ala-Asp) box helicase 17	-21.36	0.0001	0.0237
DDX5	DEAD (Asp-Glu-Ala-Asp) box helicase 5	-13.79	0.0002	0.0238
DDX52	DEAD (Asp-Glu-Ala-Asp) box polypeptide 52	-10.72	0.0007	0.0322
DEGS1	delta(4)-desaturase, sphingolipid 1	-13.79	0.0004	0.0269
DIRC2	disrupted in renal carcinoma 2	-10.08	0.0006	0.0299
DLG1	discs, large homolog 1 (Drosophila)	-14.87	0.0002	0.0243
DNAJC10	DnaJ (Hsp40) homolog, subfamily C, member 10	-5.61	0.0009	0.0346
DNAJC15	DnaJ (Hsp40) homolog, subfamily C, member 15	-21.26	5.10E-05	0.0237
DNTTIP1	deoxynucleotidyltransferase, terminal, interacting protein 1	-6.21	0.0009	0.0345
DPF2	D4, zinc and double PHD fingers family 2	-17.38	0.0005	0.0294
DPM1	dolichyl-phosphate mannosyltransferase polypeptide 1, catalytic subunit	-14.24	0.0006	0.0309
DR1	down-regulator of transcription 1	-12.43	0.0003	0.0251
DRAM2	DNA-damage regulated autophagy modulator 2	-11.89	0.0004	0.0269
DRG1	developmentally regulated GTP binding protein 1	-10.17	0.0003	0.0256
DSG2	desmoglein 2	-13.74	0.0004	0.0271
DSTN	destrin (actin depolymerizing factor)	-9.36	0.0005	0.0294
DUSP11	dual specificity phosphatase 11	-15.02	0.0004	0.0269
DUSP7	dual specificity phosphatase 7	-7.91	0.0004	0.0265

DYM	dymeclin	-12.93	0.0001	0.0237
DYNLT1	dynein, light chain, Tctex-type 1	-4.79	0.0009	0.0335
ECE1	endothelin converting enzyme 1	-28.73	7.35E-05	0.0237
EDF1	endothelial differentiation-related factor 1	-12.06	0.0003	0.0248
EEF1G	eukaryotic translation elongation factor 1 gamma	-72.28	8.69E-05	0.0237
EEF2	eukaryotic translation elongation factor 2	-35.07	4.29E-05	0.0237
EFCAB14	EF-hand calcium binding domain 14	-17.47	0.0001	0.0237
EI24	etoposide induced 2.4	-12.9	0.0005	0.0296
EIF1	eukaryotic translation initiation factor 1	-23.05	4.44E-05	0.0237
EIF2AK1	eukaryotic translation initiation factor 2-alpha kinase 1	-13.1	0.0009	0.0340
EIF2S1	eukaryotic translation initiation factor 2, subunit 1 alpha, 35kDa	-9.49	0.0006	0.0306
EIF2S3	eukaryotic translation initiation factor 2, subunit 3 gamma, 52kDa	-22.7	9.97E-05	0.0237
EIF3C	eukaryotic translation initiation factor 3, subunit C; eukaryotic translation initiation factor 3, subunit C-like	-15.95	0.0004	0.0269
EIF3D	eukaryotic translation initiation factor 3, subunit D	-33.94	6.89E-05	0.0237
EIF3H	eukaryotic translation initiation factor 3, subunit H	-10.54	0.0004	0.0272
EIF3I	eukaryotic translation initiation factor 3, subunit I	-34.66	2.48E-05	0.0237
EIF3K	eukaryotic translation initiation factor 3, subunit K	-10.51	0.0002	0.0243
EIF3M	eukaryotic translation initiation factor 3, subunit M	-33.8	5.94E-05	0.0237
EIF4A1	eukaryotic translation initiation factor 4A1; SENP3-EIF4A1 readthrough (NMD candidate)	-26.68	0.0002	0.0238
EIF4A2	eukaryotic translation initiation factor 4A2	-11.12	0.0006	0.0306
EIF4A3	eukaryotic translation initiation factor 4A3	-10.49	0.0003	0.0261
EIF4EBP2	eukaryotic translation initiation factor 4E binding protein 2	-13.33	0.0003	0.0261
EIF4G2	eukaryotic translation initiation factor 4 gamma, 2	-40.59	2.97E-05	0.0237
EIF4H	eukaryotic translation initiation factor 4H	-22.75	0.0008	0.0331
EIF4H	eukaryotic translation initiation factor 4H	-19.35	0.0009	0.0337
EMP3	epithelial membrane protein 3	-30.58	7.45E-05	0.0237
ENO1	enolase 1, (alpha)	-58.78	5.80E-05	0.0237
ENY2	enhancer of yellow 2 homolog (Drosophila)	-5.74	0.0008	0.0331
ERAL1	Era-like 12S mitochondrial rRNA chaperone 1	-16.31	0.0008	0.0331
ERBB2	erb-b2 receptor tyrosine kinase 2	-28.23	6.99E-05	0.0237
ERBB2IP	erbb2 interacting protein	-16.76	0.0005	0.0277
ERH	enhancer of rudimentary homolog (Drosophila)	-56.92	0.0002	0.0238
ERO1A	endoplasmic reticulum oxidoreductase alpha	-13.25	0.0004	0.0269
ERP44	endoplasmic reticulum protein 44	-5.86	0.0006	0.0309
ESYT1	extended synaptotagmin-like protein 1	-13.17	0.0007	0.0317
EZR	ezrin	-34.49	0.0004	0.0269

FAM120AOS	family with sequence similarity 120A opposite strand	-5.02	0.0008	0.0331
FAM20C	family with sequence similarity 20, member C	-7.63	0.0007	0.0319
FAM50A	family with sequence similarity 50, member A	-9.31	0.0006	0.0306
FARSB	phenylalanyl-tRNA synthetase beta subunit	-18.26	0.0009	0.0339
FAU; SYVN1	Finkel-Biskis-Reilly murine sarcoma virus (FBR-MuSV) ubiquitously expressed; synovial apoptosis inhibitor 1, synoviolin	-8.09	0.0010	0.0347
FBXO3	F-box protein 3	-8.06	0.0004	0.0269
FLII	flightless I actin binding protein	-13.16	0.0001	0.0237
FLNA	filamin A, alpha	-28.34	0.0008	0.0330
FMNL2	formin like 2	-7.72	0.0006	0.0305
FNBP1	formin binding protein 1	-7.55	0.0004	0.0265
FNBP4	formin binding protein 4	-6.47	0.0007	0.0312
FNTA	farnesyltransferase, CAAX box, alpha	-6.78	0.0004	0.0271
FOPNL	FGFR1OP N-terminal like	-16.76	0.0002	0.0238
FOXO3B	forkhead box O3B pseudogene	-24.51	4.44E-05	0.0237
FSTL1	follistatin like 1; microRNA 198	-11.71	0.0006	0.0309
FTH1	ferritin, heavy polypeptide 1	-5.7	0.0007	0.0319
FXYD5	FXYD domain containing ion transport regulator 5	-6.07	0.0008	0.0331
GABARAP	GABA(A) receptor-associated protein	-106.43	5.43E-05	0.0237
GABARAPL2	GABA(A) receptor-associated protein like 2	-17.65	0.0001	0.0237
GAPDH	glyceraldehyde-3-phosphate dehydrogenase	-44.94	0.0004	0.0269
GARS	glycyl-tRNA synthetase	-10.49	0.0003	0.0252
GATAD2B	GATA zinc finger domain containing 2B	-9.29	0.0003	0.0256
GBAS	glioblastoma amplified sequence	-18.24	0.0004	0.0269
GDI1	GDP dissociation inhibitor 1	-21.35	0.0001	0.0237
GDI2	GDP dissociation inhibitor 2	-22.61	0.0002	0.0238
GINM1	glycoprotein integral membrane 1	-17.26	0.0009	0.0335
GINS1	GINS complex subunit 1 (Psf1 homolog)	-25.01	0.0001	0.0237
GIT1	G protein-coupled receptor kinase interacting ArfGAP 1	-8.14	0.0006	0.0299
GLG1	golgi glycoprotein 1	-6.61	0.0008	0.0331
GLUD1	glutamate dehydrogenase 1	-14.22	0.0002	0.0243
GMPS	guanine monophosphate synthase	-21.13	0.0003	0.0261
GNAI2	guanine nucleotide binding protein (G protein), alpha inhibiting activity polypeptide 2	-6.95	0.0006	0.0305
GNB1	guanine nucleotide binding protein (G protein), beta polypeptide 1	-63.53	7.40E-05	0.0237
GNB2	guanine nucleotide binding protein (G protein), beta polypeptide 2	-6.55	0.0009	0.0340
GNB2L1	guanine nucleotide binding protein (G protein), beta polypeptide 2-like 1	-159.44	2.87E-05	0.0237
GNG11	guanine nucleotide binding protein (G protein), gamma 11	-29.9	6.99E-05	0.0237

GNG12	guanine nucleotide binding protein (G protein), gamma 12	-36.26	0.0002	0.0243
GNG5P2	guanine nucleotide binding protein (G protein), gamma 5 pseudogene 2	-6.56	0.0008	0.0333
GOLM1	golgi membrane protein 1	-8.4	0.0007	0.0319
GOT2	glutamic-oxaloacetic transaminase 2, mitochondrial	-16.11	0.0002	0.0243
GPBP1	GC-rich promoter binding protein 1	-15.65	0.0001	0.0237
GSK3A	glycogen synthase kinase 3 alpha	-5.94	0.0009	0.0340
GSTP1	glutathione S-transferase pi 1	-17.5	0.0004	0.0265
GTF2A2	general transcription factor IIA 2	-18.34	0.0002	0.0238
GTF2H1	general transcription factor IIH subunit 1	-9.9	0.0002	0.0243
GTF2H3	general transcription factor IIH subunit 3	-5.84	0.0008	0.0331
HADHA	hydroxyacyl-CoA dehydrogenase/3-ketoacyl-CoA thiolase/enoyl-CoA hydratase (trifunctional protein), alpha subunit	-14.46	0.0005	0.0278
HAUS1	HAUS augmin like complex subunit 1	-8.74	0.0003	0.0256
HAUS2	HAUS augmin like complex subunit 2	-5.04	0.0008	0.0330
HDGF	hepatoma-derived growth factor	-17.29	0.0002	0.0238
HDGFRP3	hepatoma-derived growth factor, related protein 3	-14.28	0.0004	0.0276
HDLBP	high density lipoprotein binding protein	-20.82	0.0010	0.0351
HEBP2	heme binding protein 2	-40.82	5.42E-05	0.0237
HERPUD1	homocysteine-inducible, endoplasmic reticulum stress-inducible, ubiquitin-like domain member 1	-12.74	0.0003	0.0255
HIF1A	hypoxia inducible factor 1, alpha subunit (basic helix-loop-helix transcription factor)	-39.12	0.0003	0.0250
HINT2	histidine triad nucleotide binding protein 2	-13.1	0.0003	0.0248
HIST1H1E	histone cluster 1, H1e	-86.8	0.0009	0.0340
HIST1H3D	histone cluster 1, H3d; histone cluster 1, H2ad	-10.15	0.0005	0.0295
HIST1H4C	histone cluster 1, H4c	-34.28	2.42E-05	0.0237
HIST2H2AB	histone cluster 2, H2ab	-23.3	0.0003	0.0250
HIST2H3A	histone cluster 2, H3a; histone cluster 2, H3c	-10.93	0.0005	0.0277
HIST2H3C	histone cluster 2, H3a; histone cluster 2, H3c	-10.93	0.0005	0.0277
HIST2H3PS2	histone cluster 2, H3a; histone cluster 2, H3c; histone cluster 2, H3, pseudogene 2	-21.18	9.57E-05	0.0237
HIST2H3D	histone cluster 2, H3a; histone cluster 2, H3d; histone cluster 2, H3c; histone cluster 2, H3, pseudogene 2	-31.45	4.23E-05	0.0237
HIVEP2	human immunodeficiency virus type I enhancer binding protein 2	-7.76	0.0009	0.0333
HLTF	helicase-like transcription factor	-11.04	0.0001	0.0237
HNRNPA2B1	heterogeneous nuclear ribonucleoprotein A2/B1	-6.24	0.0008	0.0331
HNRNPK	heterogeneous nuclear ribonucleoprotein K	-19.85	7.39E-05	0.0237
HOOK3	hook microtubule-tethering protein 3	-8.4	0.0010	0.0351

HP1BP3	heterochromatin protein 1, binding protein 3	-15.07	0.0001	0.0237
HSP90AA1	heat shock protein 90kDa alpha (cytosolic), class A member 1	-38.24	0.0006	0.0307
HSP90AB1	heat shock protein 90kDa alpha (cytosolic), class B member 1	-47.33	0.0007	0.0313
HSP90B1	heat shock protein 90kDa beta (Grp94), member 1; microRNA 3652	-31.42	7.04E-05	0.0237
HSPA13	heat shock protein 70kDa family, member 13	-5.23	0.0007	0.0309
HSPA5	heat shock 70kDa protein 5 (glucose-regulated protein, 78kDa)	-25.68	6.29E-05	0.0237
HSPA8	heat shock 70kDa protein 8	-129.49	0.0004	0.0271
HSPA9	heat shock 70kDa protein 9 (mortalin)	-25.68	6.04E-05	0.0237
IARS	isoleucyl-tRNA synthetase	-20.1	0.0002	0.0243
IDE	insulin-degrading enzyme	-12.26	0.0001	0.0237
IDSP1	iduronate 2-sulfatase pseudogene 1	-6.47	0.0007	0.0319
IER3	immediate early response 3	-30.6	0.0001	0.0237
IER3	immediate early response 3	-30.6	0.0001	0.0237
IER3	immediate early response 3	-31.08	0.0001	0.0237
IL6ST	interleukin 6 signal transducer	-17.15	0.0001	0.0237
ILF2	interleukin enhancer binding factor 2	-53.74	2.87E-05	0.0237
IMPAD1	inositol monophosphatase domain containing 1	-23.38	0.0002	0.0238
INTS3	integrator complex subunit 3	-6.22	0.0004	0.0272
IPO5	importin 5	-11.91	0.0006	0.0306
IPO7	importin 7	-27.44	0.0001	0.0237
IQGAP1	IQ motif containing GTPase activating protein 1	-22.09	0.0002	0.0243
IST1	increased sodium tolerance 1 homolog (yeast)	-14.47	0.0001	0.0237
ITGA3	integrin alpha 3	-34.67	3.39E-05	0.0237
ITGA6	integrin alpha 6	-17.38	0.0008	0.0331
ITGB5	integrin beta 5	-6.5	0.0006	0.0303
ITM2B	integral membrane protein 2B	-15.58	0.0003	0.0252
ITPR3	inositol 1,4,5-trisphosphate receptor, type 3	-15.69	0.0004	0.0274
JAK1	Janus kinase 1	-12.14	0.0004	0.0269
JMY	junction mediating and regulatory protein, p53 cofactor	-5.99	0.0005	0.0282
JOSD1	Josephin domain containing 1	-15.64	0.0001	0.0237
KCMF1	potassium channel modulatory factor 1	-15.21	0.0006	0.0306
KDELR1	KDEL (Lys-Asp-Glu-Leu) endoplasmic reticulum protein retention receptor 1	-12.4	0.0004	0.0269
KDELR2	KDEL (Lys-Asp-Glu-Leu) endoplasmic reticulum protein retention receptor 2; diacylglycerol lipase, beta	-33.73	6.40E-05	0.0237
KIAA1551	KIAA1551	-45.87	0.0005	0.0294
KIF5B	kinesin family member 5B	-20.53	0.0007	0.0317
KLC1	kinesin light chain 1	-5.29	0.0008	0.0331
KLF3	Kruppel-like factor 3 (basic)	-13.52	0.0001	0.0238

KMT5A	lysine (K)-specific methyltransferase 5A	-19.77	0.0002	0.0238
KMT5A	lysine (K)-specific methyltransferase 5A	-8.53	0.0006	0.0302
KPNB1	karyopherin (importin) beta 1	-27.95	0.0003	0.0262
KRT18	keratin 18, type I	-12.44	0.0005	0.0292
LAMC1	laminin, gamma 1 (formerly LAMB2)	-15.34	0.0002	0.0247
LAMP2	lysosomal-associated membrane protein 2	-6.24	0.0007	0.0309
LAPTM4A	lysosomal protein transmembrane 4 alpha	-127.05	5.05E-05	0.0237
LDHA	lactate dehydrogenase A	-164.24	2.27E-05	0.0237
LGALS1	lectin, galactoside-binding, soluble, 1	-22.17	0.0007	0.0325
LGMN	legumain	-11.13	0.0005	0.0283
LIMS1	LIM and senescent cell antigen-like domains 1	-9.34	0.0007	0.0317
LMAN1	lectin, mannose-binding, 1	-19.36	0.0006	0.0300
LMAN2	lectin, mannose-binding 2	-49.32	1.50E-05	0.0237
LOC728323	uncharacterized LOC728323	-12.96	0.0008	0.0328
LPP	LIM domain containing preferred translocation partner in lipoma	-8.22	0.0010	0.0347
LRIF1	ligand dependent nuclear receptor interacting factor 1	-8.29	0.0003	0.0256
LRRC58	leucine rich repeat containing 58	-18.66	0.0006	0.0302
LSM12	LSM12 homolog	-40.41	0.0002	0.0238
LSM12	LSM12 homolog	-39.94	0.0002	0.0238
LUZP6	leucine zipper protein 6; myotrophin	-26.15	0.0005	0.0282
CD302	LY75-CD302 readthrough; CD302 molecule	-20.12	0.0008	0.0331
LYSMD3	LysM, putative peptidoglycan-binding, domain containing 3	-7.23	0.0009	0.0333
MAFG	v-maf avian musculoaponeurotic fibrosarcoma oncogene homolog G	-8.24	0.0007	0.0325
MALAT1	metastasis associated lung adenocarcinoma transcript 1 (non-protein coding)	-74.33	2.20E-05	0.0237
MALSU1	mitochondrial assembly of ribosomal large subunit 1	-7.87	0.0002	0.0247
MAP1LC3B2	microtubule-associated protein 1 light chain 3 beta 2	-35.31	5.56E-05	0.0237
MAP4K4	mitogen-activated protein kinase kinase kinase kinase 4	-11.31	0.0008	0.0331
MAPK1	mitogen-activated protein kinase 1	-22.38	0.0002	0.0243
MAPK3	mitogen-activated protein kinase 3	-7.61	0.0009	0.0346
MAPKAPK3	mitogen-activated protein kinase-activated protein kinase 3	-5.14	0.0008	0.0331
MARCH7	membrane associated ring finger 7	-7.37	0.0008	0.0329
MBNL1	muscleblind like splicing regulator 1	-9.37	0.0004	0.0269
MBOAT1	membrane bound O-acyltransferase domain containing 1	-11.12	0.0001	0.0237
MBOAT2	membrane bound O-acyltransferase domain containing 2	-15.67	0.0007	0.0313
MCFD2	multiple coagulation factor deficiency 2	-9.25	0.0003	0.0252
MCL1	myeloid cell leukemia 1	-12.48	0.0001	0.0237
MCU	mitochondrial calcium uniporter	-27.91	0.0002	0.0243
ME2	malic enzyme 2, NAD(+)-dependent, mitochondrial	-8.01	0.0002	0.0247

MEA1	male-enhanced antigen 1	-18.56	0.0004	0.0269
MEAF6	MYST/Esa1-associated factor 6	-6.68	0.0005	0.0297
MED13	mediator complex subunit 13	-9.62	0.0004	0.0269
MED31	mediator complex subunit 31	-6.54	0.0009	0.0333
MET	MET proto-oncogene, receptor tyrosine kinase	-17.55	7.27E-05	0.0237
METAP1	methionyl aminopeptidase 1	-9.21	0.0008	0.0331
MFSD14A	major facilitator superfamily domain containing 14A	-16.55	0.0002	0.0243
MGST3	microsomal glutathione S-transferase 3	-12.42	0.0004	0.0265
MKNK2	MAP kinase interacting serine/threonine kinase 2	-4.59	0.0010	0.0349
MORN2	MORN repeat containing 2	-6.39	0.0005	0.0281
MPV17	MpV17 mitochondrial inner membrane protein	-19.65	0.0007	0.0323
MRPL17	mitochondrial ribosomal protein L17	-10.94	0.0004	0.0271
MRPL28	mitochondrial ribosomal protein L28	-16.35	0.0004	0.0266
MRPL42	mitochondrial ribosomal protein L42	-9.27	0.0008	0.0326
MRPL45	mitochondrial ribosomal protein L45	-112.14	5.50E-05	0.0237
MRPL46	mitochondrial ribosomal protein L46	-7.12	0.0006	0.0307
MRPL51	mitochondrial ribosomal protein L51	-17.04	0.0002	0.0243
MRPS11	mitochondrial ribosomal protein S11	-13.13	0.0004	0.0269
MRPS18A	mitochondrial ribosomal protein S18A	-19.39	5.62E-05	0.0237
MRPS21	mitochondrial ribosomal protein S21	-18.02	6.41E-05	0.0237
MSANTD3	Myb/SANT-like DNA-binding domain containing 3; MSANTD3-TMEFF1 readthrough	-5.63	0.0006	0.0306
MSL1	male-specific lethal 1 homolog (Drosophila)	-19.44	7.52E-05	0.0237
MSN	moesin	-42.58	0.0009	0.0335
MTFR1	mitochondrial fission regulator 1	-8.42	0.0002	0.0243
MYL12B	myosin light chain 12B	-31.01	7.06E-05	0.0237
MYL6	myosin light chain 6; myosin light chain 6B	-19.65	0.0001	0.0237
NAA50	N(alpha)-acetyltransferase 50, NatE catalytic subunit	-20.13	0.0006	0.0309
NARS	asparaginyl-tRNA synthetase	-15.38	0.0009	0.0340
NARS2	asparaginyl-tRNA synthetase 2, mitochondrial (putative)	-6.05	0.0006	0.0306
NCKAP1	NCK-associated protein 1	-13.78	0.0002	0.0245
NDFIP1	Nedd4 family interacting protein 1	-4.67	0.0009	0.0339
NDRG3	NDRG family member 3	-22.51	0.0009	0.0335
NDUFA12	NADH dehydrogenase (ubiquinone) 1 alpha subcomplex, 12	-9.68	0.0002	0.0243
NDUFA2	NADH dehydrogenase (ubiquinone) 1 alpha subcomplex, 2, 8kDa	-12.87	0.0008	0.0331
NDUFB5	NADH dehydrogenase (ubiquinone) 1 beta subcomplex, 5, 16kDa	-14.64	0.0008	0.0331
NECAP2	NECAP endocytosis associated 2	-6.11	0.0006	0.0307

NEDD8	NEDD8-MDP1 readthrough; neural precursor cell expressed, developmentally down-regulated 8	-20.85	0.0003	0.0256
NEMP1	nuclear envelope integral membrane protein 1	-13.66	0.0006	0.0309
NETO2	neuropilin (NRP) and tolloid (TLL)-like 2	-42.99	0.0003	0.0252
NFE2L1	nuclear factor, erythroid 2-like 1	-14.98	0.0001	0.0237
NIFK	nucleolar protein interacting with the FHA domain of MKI67	-9.42	0.0007	0.0317
NIT2	nitrilase family, member 2	-21.78	6.89E-05	0.0237
NME1	NME/NM23 nucleoside diphosphate kinase 1; NME1-NME2 readthrough; NME/NM23 nucleoside diphosphate kinase 2	-9.18	0.0003	0.0256
NME2P1	NME/NM23 nucleoside diphosphate kinase 2 pseudogene 1	-30.84	0.0002	0.0238
NOL11	nucleolar protein 11	-10.76	0.0002	0.0243
NOMO1	NODAL modulator 1; NODAL modulator 2	-14.18	0.0005	0.0282
NPLOC4	NPL4 homolog, ubiquitin recognition factor	-18.9	0.0010	0.0347
NPTN	neuroplastin	-9.93	0.0005	0.0294
NQO1	NAD(P)H dehydrogenase, quinone 1	-17.73	0.0009	0.0346
NQO2	NAD(P)H dehydrogenase, quinone 2	-6.65	0.0003	0.0261
NSL1	NSL1, MIS12 kinetochore complex component	-9.84	0.0002	0.0243
NT5C2	5-nucleotidase, cytosolic II	-11.96	0.0007	0.0312
NUCKS1	nuclear casein kinase and cyclin-dependent kinase substrate 1	-22.95	9.65E-05	0.0237
NUDT21	nudix hydrolase 21	-16.86	0.0005	0.0293
NUDT4	nudix hydrolase 4; nudix hydrolase 4 pseudogene 2; nudix hydrolase 4 pseudogene 1	-7.62	0.0010	0.0351
NUFIP2	nuclear fragile X mental retardation protein interacting protein 2	-7.25	0.0004	0.0276
NUP153	nucleoporin 153kDa	-12.61	0.0006	0.0308
NUP155	nucleoporin 155kDa	-17.41	0.0008	0.0331
NUP88	nucleoporin 88kDa	-18.12	0.0002	0.0245
OAZ1	ornithine decarboxylase antizyme 1; signal peptide peptidase like 2B	-69.41	0.0007	0.0312
OCLN	occludin	-29.12	8.83E-05	0.0237
OGFRL1	opioid growth factor receptor-like 1	-13.1	0.0004	0.0269
OSBPL8	oxysterol binding protein-like 8	-6.29	0.0004	0.0269
OSTC	oligosaccharyltransferase complex subunit (non-catalytic)	-8.47	0.0003	0.0261
OXSR1	oxidative stress responsive 1	-6.18	0.0006	0.0309
P4HB	prolyl 4-hydroxylase, beta polypeptide	-39.01	8.19E-05	0.0237
P4HB	prolyl 4-hydroxylase, beta polypeptide	-27.25	0.0003	0.0259
PABPC1	poly(A) binding protein, cytoplasmic 1	-21.39	0.0003	0.0256
PACS1	phosphofurin acidic cluster sorting protein 1	-10.21	0.0003	0.0256
PAFAH1B1	platelet-activating factor acetylhydrolase 1b, regulatory subunit 1 (45kDa)	-12.84	0.0001	0.0237
PAIP1	poly(A) binding protein interacting protein 1	-7.17	0.0004	0.0271

PAIP2	poly(A) binding protein interacting protein 2	-8.06	0.0005	0.0279
PAK2	p21 protein (Cdc42/Rac)-activated kinase 2	-19.04	0.0004	0.0271
PAPD4	PAP associated domain containing 4	-7.76	0.0006	0.0306
PAPOLA	poly(A) polymerase alpha	-14.9	0.0003	0.0252
PARK7	parkinson protein 7	-8.8	0.0002	0.0245
PARN	poly(A)-specific ribonuclease	-6.66	0.0007	0.0325
PARP4	poly(ADP-ribose) polymerase family member 4	-15.25	0.0003	0.0252
PARVA	parvin, alpha	-34.99	3.05E-05	0.0237
PATL1	protein associated with topoisomerase II homolog 1 (yeast)	-50.04	0.0002	0.0240
PCBP2	poly(rC) binding protein 2; PCBP2 overlapping transcript 1	-48.85	1.86E-05	0.0237
PCYOX1	prenylcysteine oxidase 1	-18.05	0.0004	0.0272
PDE6D	phosphodiesterase 6D, cGMP-specific, rod, delta	-11.14	0.0002	0.0243
PDIA3	protein disulfide isomerase family A member 3	-22.73	0.0001	0.0237
PDIA3	protein disulfide isomerase family A member 3; protein disulfide isomerase family A member 3 pseudogene 1	-25.32	4.32E-05	0.0237
PDIA6	protein disulfide isomerase family A, member 6	-11.28	0.0009	0.0347
PDS5A	PDS5 cohesin associated factor A	-13.92	0.0005	0.0294
PEA15	phosphoprotein enriched in astrocytes 15	-21.21	0.0003	0.0256
PFDN1	prefoldin subunit 1	-15.24	0.0007	0.0317
PFN1	profilin 1	-32.41	0.0002	0.0238
PFN1P3	profilin 1 pseudogene 3	-79.67	0.0001	0.0237
PFN2	profilin 2	-22.05	3.99E-05	0.0237
PGK1	phosphoglycerate kinase 1	-24.6	0.0005	0.0298
PGRMC1	progesterone receptor membrane component 1	-28.77	4.84E-05	0.0237
PHACTR4	phosphatase and actin regulator 4	-26.72	9.89E-05	0.0237
PHB	prohibitin	-7.85	0.0010	0.0351
PHC3	polyhomeotic homolog 3 (Drosophila)	-8.09	0.0009	0.0340
PHGDH	phosphoglycerate dehydrogenase	-5.96	0.0006	0.0309
PHLDA1	pleckstrin homology-like domain, family A, member 1	-10.41	0.0003	0.0252
PICALM	phosphatidylinositol binding clathrin assembly protein	-21.63	5.35E-05	0.0237
PIGX	phosphatidylinositol glycan anchor biosynthesis class X	-8.34	0.0007	0.0321
PIK3C2A	phosphatidylinositol-4-phosphate 3-kinase, catalytic subunit type 2 alpha	-11.31	0.0005	0.0294
PITPNA	phosphatidylinositol transfer protein, alpha	-9.87	0.0002	0.0243
PKM	pyruvate kinase, muscle	-47.02	9.55E-05	0.0237
PKN2	protein kinase N2	-10.92	0.0007	0.0319
PLCD3	phospholipase C, delta 3	-9.39	0.0002	0.0243
PLEKHA2	pleckstrin homology domain containing, family A (phosphoinositide binding specific) member 2	-7.16	0.0004	0.0270

PLOD1	procollagen-lysine, 2-oxoglutarate 5-dioxygenase 1	-10.2	0.0003	0.0261
PLP2	proteolipid protein 2 (colonic epithelium-enriched)	-34.5	0.0003	0.0261
PLXNB2	plexin B2	-10.48	0.0003	0.0256
PMS2P2	[illegible] homolog 2, mismatch repair system component pseudogene 4; DTX2P1-UPK3BP1-PMS2P11 readthrough transcribed pseudogene; PMS1 homolog 2, mismatch repair system component pseudogene 5; PMS1 homolog 2, mismatch repair system component pseudogene 7; PMS1 homolog 2, mismatch repair system component pseudogene 10; PMS1 homolog 2, mismatch repair system component pseudogene 6	-10.19	0.0006	0.0306
PMS2P7	PMS1 homolog 2, mismatch repair system component pseudogene 2; PMS1 homolog 2, mismatch repair system component pseudogene 7; PMS1 homolog 2, mismatch repair system component pseudogene 10; PMS1 homolog 2, mismatch repair system component pseudogene 6; PMS1 homolog 2, mismatch repair system component pseudogene 5	-7.13	0.0004	0.0271
PMS2P10	PMS1 homolog 2, mismatch repair system component pseudogene 7; PMS1 homolog 2, mismatch repair system component pseudogene 10; PMS1 homolog 2, mismatch repair system component pseudogene 6; PMS1 homolog 2, mismatch repair system component pseudogene 2	-6.17	0.0008	0.0331
PMS2P6	PMS1 homolog 2, mismatch repair system component pseudogene 7; PMS1 homolog 2, mismatch repair system component pseudogene 6; PMS1 homolog 2, mismatch repair system component pseudogene 10; PMS1 homolog 2, mismatch repair system component pseudogene 2	-6.96	0.0005	0.0282
POLR2H	polymerase (RNA) II (DNA directed) polypeptide H	-22.2	0.0003	0.0256
POLR2J4	polymerase (RNA) II (DNA directed) polypeptide J4, pseudogene; polymerase (RNA) II (DNA directed) polypeptide J2	-21.62	5.14E-05	0.0237
PPA1	pyrophosphatase (inorganic) 1	-10.49	0.0002	0.0243
PPHLN1	periphilin 1	-7.91	0.0008	0.0327
PPIB	peptidylprolyl isomerase B (cyclophilin B)	-61.45	5.29E-05	0.0237
PPP1CC	protein phosphatase 1, catalytic subunit, gamma isozyme	-29.51	0.0007	0.0321
PPP1R14B	protein phosphatase 1, regulatory (inhibitor) subunit 14B	-8.34	0.0003	0.0252
PPP1R1B	protein phosphatase 1, regulatory (inhibitor) subunit 1B	-12.65	0.0006	0.0302
PPP2CB	protein phosphatase 2, catalytic subunit, beta isozyme	-7.91	0.0004	0.0269
PPP6C	protein phosphatase 6, catalytic subunit	-27.5	0.0006	0.0305
PRDX1	peroxiredoxin 1	-34.51	0.0001	0.0237
PRRC2C	proline-rich coiled-coil 2C	-14.71	0.0006	0.0309
PSAP	prosaposin	-10.34	0.0005	0.0297
PSEN1	presenilin 1	-6.76	0.0006	0.0305

PSMA1	proteasome subunit alpha 1	-28.37	0.0002	0.0238
PSMA6	proteasome subunit alpha 6; KIAA0391	-15.25	0.0002	0.0243
PSMB3	proteasome subunit beta 3	-15.15	0.0009	0.0343
PSMB4	proteasome subunit beta 4	-10.14	0.0006	0.0309
PSMB7	proteasome subunit beta 7	-20.27	0.0001	0.0237
PSMD10	proteasome 26S subunit, non-ATPase 10	-28.28	6.67E-05	0.0237
PSMD7	proteasome 26S subunit, non-ATPase 7	-8.56	0.0004	0.0268
PSMD8	proteasome 26S subunit, non-ATPase 8	-16.75	9.62E-05	0.0237
PSME3	proteasome activator subunit 3	-33.87	0.0003	0.0258
PSMF1	proteasome inhibitor subunit 1	-10.7	0.0004	0.0271
PTBP1	polypyrimidine tract binding protein 1	-36.03	0.0003	0.0252
PTK2	protein tyrosine kinase 2	-9.11	0.0006	0.0309
PTPN12	protein tyrosine phosphatase, non-receptor type 12	-9.73	0.0003	0.0256
PTPRJ	protein tyrosine phosphatase, receptor type, J	-5.15	0.0010	0.0349
PTTG1IP	pituitary tumor-transforming 1 interacting protein	-28.16	0.0006	0.0306
PUM2	pumilio RNA binding family member 2	-7.3	0.0003	0.0261
PURA	purine-rich element binding protein A	-13.72	0.0007	0.0313
PYGB	phosphorylase, glycogen; brain	-14.05	0.0003	0.0261
QRICH1	glutamine-rich 1	-17.96	0.0003	0.0252
QSER1	glutamine and serine rich 1	-14.89	0.0009	0.0342
RAB10	RAB10, member RAS oncogene family	-36.45	8.50E-05	0.0237
RAB11A	RAB11A, member RAS oncogene family	-12.77	0.0008	0.0330
RAB21	RAB21, member RAS oncogene family	-12.78	0.0001	0.0237
RAB2A	RAB2A, member RAS oncogene family	-5.68	0.0005	0.0282
RAB31	RAB31, member RAS oncogene family	-9.15	0.0003	0.0262
RAB5C	RAB5C, member RAS oncogene family	-45.74	0.0007	0.0316
RAB6C	RAB6C, member RAS oncogene family	-13.98	0.0002	0.0245
RAB6C	RAB6C, member RAS oncogene family	-5.13	0.0007	0.0316
RAB7A	RAB7A, member RAS oncogene family	-99.71	7.72E-05	0.0237
RABGAP1	RAB GTPase activating protein 1	-6.05	0.0010	0.0351
RABL2B	RAB, member of RAS oncogene family-like 2B	-5.91	0.0009	0.0345
RABL2B	RAB, member of RAS oncogene family-like 2B; RAB, member of RAS oncogene family-like 2A	-8	0.0004	0.0271
RAD23B	RAD23 homolog B, nucleotide excision repair protein	-5.41	0.0006	0.0308
RALY	RALY heterogeneous nuclear ribonucleoprotein	-10.06	0.0008	0.0331
RANBP9	RAN binding protein 9	-10.75	0.0004	0.0271
RANP1	RAN, member RAS oncogene family pseudogene 1	-11.08	0.0002	0.0238
RAP1B	RAP1B, member of RAS oncogene family	-22.32	0.0003	0.0261

RAP1B	RAP1B, member of RAS oncogene family	-7.92	0.0005	0.0294
RARS	arginyl-tRNA synthetase	-11.33	0.0001	0.0237
RB1	retinoblastoma 1	-8.66	0.0008	0.0331
RBBP4	retinoblastoma binding protein 4	-27.63	0.0009	0.0339
RBBP7	retinoblastoma binding protein 7	-17.1	0.0002	0.0245
RBBP8	retinoblastoma binding protein 8	-17.36	0.0001	0.0237
RBMS1	RNA binding motif, single stranded interacting protein 1	-10.79	0.0005	0.0294
RBMY1B	RNA binding motif protein, Y-linked, family 1, member B; RNA binding motif protein, Y-linked, family 1, member D; RNA binding motif protein, Y-linked, family 1, member E	4.67	0.0008	0.0331
REEP3	receptor accessory protein 3	-7.61	0.0009	0.0339
REEP5	receptor accessory protein 5	-39.79	0.0001	0.0237
REST	RE1-silencing transcription factor	-5.48	0.0008	0.0328
RFC1	replication factor C subunit 1	-8.1	0.0010	0.0351
RFWD3	ring finger and WD repeat domain 3	-21.3	0.0002	0.0238
RHOA	ras homolog family member A	-40.74	0.0004	0.0270
RHOF	ras homolog family member F (in filopodia)	-9.83	0.0009	0.0339
RHOT1	ras homolog family member T1	-7.15	0.0007	0.0312
RIN2	Ras and Rab interactor 2	-6.98	0.0009	0.0340
RNF130	ring finger protein 130	-6.83	0.0005	0.0282
RNF43	ring finger protein 43; SPT4 homolog, DSIF elongation factor subunit	-10.55	0.0006	0.0306
RPAP3	RNA polymerase II associated protein 3	-8.86	0.0002	0.0243
RPL10	ribosomal protein L10	-122.74	0.0002	0.0239
RPL13AP5	ribosomal protein L13a pseudogene 5; ribosomal protein L13a	-35.75	0.0006	0.0302
RPL15	ribosomal protein L15; ribosomal protein L15 pseudogene 3	-21.8	0.0002	0.0238
RPL18	ribosomal protein L18	-80.96	0.0006	0.0306
RPL28	ribosomal protein L28	-7.89	0.0002	0.0243
RPL30	ribosomal protein L30	-85.19	1.29E-05	0.0237
RPL31	ribosomal protein L31	-4.8	0.0010	0.0351
RPL35	ribosomal protein L35	-36.37	4.50E-05	0.0237
RPL35A	ribosomal protein L35a	-30.54	0.0004	0.0269
RPL8	ribosomal protein L8	-46.58	0.0002	0.0238
RPLP2	ribosomal protein, large, P2	-84.9	0.0001	0.0237
RPN1	ribophorin I	-10.83	0.0005	0.0294
RPS11	ribosomal protein S11	-46.45	0.0002	0.0238
RPS11P5	ribosomal protein S11 pseudogene 5	-17.65	0.0002	0.0243
RPS14	ribosomal protein S14	-51.51	0.0006	0.0307
RPS15AP1	ribosomal protein S15a pseudogene 1	-15.07	0.0008	0.0332

RPS16	ribosomal protein S16	-9.76	0.0003	0.0256
RPS17	ribosomal protein S17	-12.64	0.0002	0.0243
RPS17	ribosomal protein S17	-12.64	0.0002	0.0243
RPS19	ribosomal protein S19	-10.8	0.0002	0.0238
RPS23	ribosomal protein S23	-8.54	0.0008	0.0331
RPS3	ribosomal protein S3	-76.43	5.25E-05	0.0237
RPS4X	ribosomal protein S4, X-linked	-8.17	0.0007	0.0325
RPS5	ribosomal protein S5	-8.8	0.0004	0.0269
RQCD1	RCD1 required for cell differentiation1 homolog (S. pombe)	-12.27	0.0004	0.0271
RYK	receptor-like tyrosine kinase	-27.04	5.34E-05	0.0237
S100A10	S100 calcium binding protein A10	-63.31	0.0002	0.0243
S100A11	S100 calcium binding protein A11	-43.58	0.0007	0.0309
S100A16	S100 calcium binding protein A16	-25.09	0.0004	0.0269
SARAF	store-operated calcium entry-associated regulatory factor	-5.61	0.0007	0.0321
SARS	seryl-tRNA synthetase	-18.02	0.0004	0.0269
SCAF11	SR-related CTD-associated factor 11	-5.44	0.0006	0.0306
SCAMP1	secretory carrier membrane protein 1	-5.28	0.0008	0.0331
SDCBP	syndecan binding protein	-12.63	0.0006	0.0306
SDHB	succinate dehydrogenase complex subunit B, iron sulfur (Ip)	-10.27	0.0009	0.0342
SEC23A	Sec23 homolog A, COPII coat complex component	-12.87	0.0001	0.0237
SEC61A1	Sec61 translocon alpha 1 subunit	-15.95	0.0006	0.0304
SEC63	SEC63 homolog, protein translocation regulator	-5.54	0.0008	0.0326
SEMA4B	sema domain, immunoglobulin domain (Ig), transmembrane domain (TM) and short cytoplasmic domain, (semaphorin) 4B	-6.57	0.0007	0.0321
SENP6	SUMO1/sentrin specific peptidase 6	-6.05	0.0008	0.0331
SERINC3	serine incorporator 3	-30.02	0.0006	0.0309
SERP1	stress-associated endoplasmic reticulum protein 1	-12.86	0.0002	0.0245
SERPINB1	serpin peptidase inhibitor, clade B (ovalbumin), member 1	-10.6	0.0001	0.0237
SET	SET nuclear proto-oncogene	-84.42	0.0002	0.0243
SF3A3	splicing factor 3a subunit 3	-14.52	0.0007	0.0311
SF3B1	splicing factor 3b, subunit 1, 155kDa	-28.42	3.91E-05	0.0237
SGF29	SAGA complex associated factor 29	-8.24	0.0004	0.0271
SGPL1	sphingosine-1-phosphate lyase 1	-6.27	0.0008	0.0331
SIPA1L1	signal-induced proliferation-associated 1 like 1	-5.76	0.0008	0.0331
SLBP	stem-loop binding protein	-6.06	0.0007	0.0319
SLC20A1	solute carrier family 20 (phosphate transporter), member 1	-13.61	0.0001	0.0237
SLC25A6	solute carrier family 25 (mitochondrial carrier; adenine nucleotide translocator), member 6	-24.45	0.0007	0.0312

SLC25A6	solute carrier family 25 (mitochondrial carrier; adenine nucleotide translocator), member 6	-24.45	0.0007	0.0312
SLC2A1	solute carrier family 2 (facilitated glucose transporter), member 1	-40.33	6.56E-05	0.0237
SLC35B1	solute carrier family 35, member B1	-6.9	0.0003	0.0256
SLC35F5	solute carrier family 35, member F5	-7.5	0.0005	0.0277
SLC38A1	solute carrier family 38, member 1	-27.77	7.24E-05	0.0237
SLC41A2	solute carrier family 41 (magnesium transporter), member 2	-5.42	0.0009	0.0340
SLC44A1	solute carrier family 44 (choline transporter), member 1	-8.33	0.0002	0.0243
SLC7A5	solute carrier family 7 (amino acid transporter light chain, L system), member	-23.55	0.0003	0.0250
SLCO1B3	solute carrier organic anion transporter family, member 1B3	-7.69	0.0008	0.0331
SLFN11	schlafen family member 11	-12.93	0.0007	0.0319
SMAD2	SMAD family member 2	-6.99	0.0006	0.0309
SMAD3	SMAD family member 3	-14.22	0.0006	0.0309
SMAD4	SMAD family member 4	-11.27	0.0001	0.0237
SMG1	SMG1 phosphatidylinositol 3-kinase-related kinase	-17.76	0.0009	0.0333
SMN2; SMN1	survival of motor neuron 2, centromeric; survival of motor neuron 1, telomeric	-7.51	0.0004	0.0272
SMNDC1	survival motor neuron domain containing 1	-11.92	0.0001	0.0237
SMURF2	SMAD specific E3 ubiquitin protein ligase 2	-12.97	0.0003	0.0261
SNAI2	snail family zinc finger 2	-24.33	0.0009	0.0343
SNORA61	small nucleolar RNA, H/ACA box 61; small nucleolar RNA host gene 12	-10.55	0.0003	0.0262
RPL10	small nucleolar RNA, H/ACA box 70; ribosomal protein L10	-6.97	0.0005	0.0295
SNORD14C	small nucleolar RNA, C/D box 14C; heat shock 70kDa protein 8	-26.2	0.0005	0.0294
SNORD41	small nucleolar RNA, C/D box 41	-14.33	0.0003	0.0256
SNRNP27	small nuclear ribonucleoprotein, U4/U6.U5 27kDa subunit; MAX dimerization protein 1	-13.61	0.0001	0.0237
SNRNP48	small nuclear ribonucleoprotein, U11/U12 48KDa subunit	-6.7	0.0004	0.0271
SNRPB2	small nuclear ribonucleoprotein polypeptide B	-7.7	0.0006	0.0309
SNX3	sorting nexin 3	-8.94	0.0003	0.0252
SNX6	sorting nexin 6	-10.41	0.0003	0.0256
SOS1	SOS Ras/Rac guanine nucleotide exchange factor 1	-9.26	0.0002	0.0243
SP1	Sp1 transcription factor	-11.99	0.0006	0.0306
SPA17	sperm autoantigenic protein 17	-6.15	0.0010	0.0351
SPCS2	signal peptidase complex subunit 2	-7.44	0.0009	0.0340
SPCS3	signal peptidase complex subunit 3	-9.74	0.0003	0.0252
SPIN1	spindlin 1	-31.58	0.0002	0.0243
SPIRE1	spire-type actin nucleation factor 1	-6.27	0.0006	0.0306
SPPL2A	signal peptide peptidase like 2A	-19.61	6.06E-05	0.0237
SRI	sorcin	-29.8	4.52E-05	0.0237

SRP54	signal recognition particle 54kDa	-5	0.0009	0.0343
SRPK1	SRSF protein kinase 1	-16.12	0.0001	0.0237
SRRM2	serine/arginine repetitive matrix 2	-13.66	0.0003	0.0250
SSR2	signal sequence receptor, beta (translocon-associated protein beta)	-39.03	0.0001	0.0238
SSR3	signal sequence receptor, gamma (translocon-associated protein gamma)	-16.3	0.0008	0.0331
STARD3	StAR-related lipid transfer domain containing 3	-13.39	0.0003	0.0261
STAT3	signal transducer and activator of transcription 3 (acute-phase response factor)	-16.2	0.0004	0.0271
STK24	serine/threonine kinase 24	-8.89	0.0005	0.0278
STK38	serine/threonine kinase 38	-10.44	0.0008	0.0332
STOML2	stomatin (EPB72)-like 2	-15.65	0.0003	0.0261
STRADB	STE20-related kinase adaptor beta	-11.82	0.0001	0.0237
STT3B	STT3B, subunit of the oligosaccharyltransferase complex (catalytic)	-20.12	0.0001	0.0237
STX2	syntaxin 2	-7.55	0.0008	0.0330
SUB1	SUB1 homolog, transcriptional regulator	-5.34	0.0006	0.0306
SULF2	sulfatase 2	-9.51	0.0005	0.0282
SURF4	surfeit 4	-9.87	0.0006	0.0306
SUZ12	SUZ12 polycomb repressive complex 2 subunit	-17.62	5.75E-05	0.0237
COX16	SYNJ2BP-COX16 readthrough; COX16 cytochrome c oxidase assembly homolog	-28.38	0.0004	0.0265
SYPL1	synaptophysin-like 1	-9.46	0.0002	0.0238
SZRD1	SUZ RNA binding domain containing 1	-16.82	0.0001	0.0237
TAF10	TAF10 RNA polymerase II, TATA box binding protein (TBP)-associated factor, 30kDa	-36.05	2.74E-05	0.0237
TAF1D	TATA box binding protein associated factor 1D; small nucleolar RNA, H/ACA box 25; small nucleolar RNA, H/ACA box 8; small nucleolar RNA, H/ACA box 18	-5.34	0.0006	0.0308
TALDO1	transaldolase 1	-6.41	0.0004	0.0269
TAOK1	TAO kinase 1	-11.13	0.0006	0.0309
TBL1XR1	transducin (beta)-like 1 X-linked receptor 1	-8.95	0.0002	0.0243
TBRG1	transforming growth factor beta regulator 1	-5.51	0.0006	0.0309
TCEA1	transcription elongation factor A (SII), 1	-22.57	5.94E-05	0.0237
TCEA1	transcription elongation factor A (SII), 1	-17.81	0.0003	0.0262
TCEB2	transcription elongation factor B (SIII), polypeptide 2 (18kDa, elongin B)	-16.48	0.0003	0.0261
TCF7L2	transcription factor 7-like 2 (T-cell specific, HMG-box)	-7.72	0.0005	0.0294
TCP1	t-complex 1	-48.44	0.0004	0.0269
TES	testin LIM domain protein	-17.02	0.0002	0.0238
TFB2M	transcription factor B2, mitochondrial	-6.33	0.0008	0.0333
TFDP1	transcription factor Dp-1	-30.87	0.0002	0.0238

TFG	TRK-fused gene	-13.44	0.0003	0.0261
TGFBRAP1	transforming growth factor beta receptor associated protein 1	-6.53	0.0006	0.0305
TGOLN2	trans-golgi network protein 2	-6.19	0.0009	0.0339
TIA1; C2orf42	TIA1 cytotoxic granule-associated RNA binding protein; chromosome 2 open reading frame 42	-11.22	0.0004	0.0269
TIMM23	translocase of inner mitochondrial membrane 23 homolog (yeast); translocase of inner mitochondrial membrane 23 homolog B (yeast)	-16.75	0.0003	0.0256
TIMM23B	translocase of inner mitochondrial membrane 23 homolog (yeast); translocase of inner mitochondrial membrane 23 homolog B (yeast)	-16.71	0.0004	0.0269
TIMM50	translocase of inner mitochondrial membrane 50 homolog (S. cerevisiae)	-34.91	0.0002	0.0238
TKT	transketolase	-12.18	0.0010	0.0351
TM9SF2	transmembrane 9 superfamily member 2	-39.82	0.0002	0.0247
TMBIM1	transmembrane BAX inhibitor motif containing 1	-13.05	0.0008	0.0331
TMBIM6	transmembrane BAX inhibitor motif containing 6	-57.41	2.96E-05	0.0237
TMED10	transmembrane p24 trafficking protein 10	-36.17	8.78E-05	0.0237
TMED2	transmembrane p24 trafficking protein 2	-49.07	2.73E-05	0.0237
TMED4	transmembrane p24 trafficking protein 4	-5.69	0.0005	0.0290
TMED7	TMED7-TICAM2 readthrough; toll-like receptor adaptor molecule 2; transmembrane p24 trafficking protein 7	-4.93	0.0007	0.0321
TMEM106C	transmembrane protein 106C	-14.08	0.0004	0.0269
TMEM123	transmembrane protein 123	-19.52	7.95E-05	0.0237
TMEM141	transmembrane protein 141	-7.36	0.0004	0.0269
TMEM147	transmembrane protein 147	-13.44	0.0010	0.0351
TMEM14B	transmembrane protein 14B	-55.89	5.46E-05	0.0237
TMEM14C	transmembrane protein 14C	-23.92	0.0008	0.0331
TMEM209	transmembrane protein 209	-11.89	0.0005	0.0290
TMEM223	transmembrane protein 223	-9.28	0.0008	0.0329
TMEM230	transmembrane protein 230	-10.5	0.0007	0.0321
TMEM243	transmembrane protein 243, mitochondrial	-7.61	0.0008	0.0331
CTNNAL1	transmembrane protein 245; catenin (cadherin-associated protein), alpha-like 1	-10.36	0.0006	0.0305
TMEM248	transmembrane protein 248	-20.94	0.0002	0.0238
COX7A2	transmembrane protein 30A; cytochrome c oxidase subunit VIIa polypeptide 2 (liver)	-5.33	0.0010	0.0351
TMEM50A	transmembrane protein 50A	-25.47	0.0001	0.0237
TMEM59	transmembrane protein 59	-10.87	0.0005	0.0294
TMSB10	thymosin beta 10	-94.99	4.05E-05	0.0237
TNFAIP1	tumor necrosis factor, alpha-induced protein 1 (endothelial)	-8.55	0.0003	0.0256
TNFRSF10B	tumor necrosis factor receptor superfamily, member 10b	-6.77	0.0010	0.0351

TOM1L1	target of myb1 like 1 membrane trafficking protein	-11.66	0.0006	0.0302
TOMM34	translocase of outer mitochondrial membrane 34	-11.22	0.0008	0.0331
TOP1	topoisomerase (DNA) I	-12.59	0.0002	0.0243
TOR1AIP2	torsin A interacting protein 2	-13.52	0.0002	0.0243
TPD52L2	tumor protein D52-like 2	-7.28	0.0003	0.0258
TPGS2	tubulin polyglutamylase complex subunit 2	-17.99	0.0003	0.0256
TRAM1	translocation associated membrane protein 1	-8.54	0.0003	0.0248
TRIM25	tripartite motif containing 25; microRNA 3614	-7.83	0.0003	0.0255
TRIM33	tripartite motif containing 33	-17.33	0.0005	0.0281
RPP21	TRIM39-RPP21 readthrough; ribonuclease P/MRP 21kDa subunit	-10.07	0.0009	0.0339
TRIP12	thyroid hormone receptor interactor 12	-7.96	0.0008	0.0331
TRMT112	tRNA methyltransferase 11-2 homolog (S. cerevisiae)	-59.09	0.0003	0.0256
TSPAN1	tetraspanin 1	-7.24	0.0004	0.0269
TSPAN31	tetraspanin 31	-5.04	0.0008	0.0331
TUG1	taurine up-regulated 1 (non-protein coding)	-36.45	0.0002	0.0238
TWF2	twinfilin actin binding protein 2	-11.91	0.0001	0.0237
TXNDC12	thioredoxin domain containing 12 (endoplasmic reticulum)	-19.13	0.0008	0.0331
TXNL1	thioredoxin-like 1	-7.11	0.0003	0.0259
U2AF1	U2 small nuclear RNA auxiliary factor 1; U2 small nuclear RNA auxiliary factor 1-like 5	-12.27	0.0001	0.0237
UBA2	ubiquitin-like modifier activating enzyme 2	-20.56	0.0001	0.0237
UBBP4	ubiquitin B pseudogene 4	-11.26	0.0009	0.0339
UBE2A	ubiquitin conjugating enzyme E2A	-21.19	0.0001	0.0237
UBE2K	ubiquitin conjugating enzyme E2K	-23.91	0.0001	0.0237
UBE2Q1	ubiquitin-conjugating enzyme E2Q family member 1	-11.75	0.0002	0.0240
UBE2R2	ubiquitin-conjugating enzyme E2R 2	-18.58	6.78E-05	0.0237
UBE2Z	ubiquitin-conjugating enzyme E2Z	-27.65	3.89E-05	0.0237
UBP1	upstream binding protein 1 (LBP-1a)	-8.95	0.0005	0.0277
UBQLN1	ubiquilin 1	-16.44	8.91E-05	0.0237
UBR4	ubiquitin protein ligase E3 component n-recognin 4	-11.69	0.0009	0.0339
UBXN7	UBX domain protein 7	-14.3	8.98E-05	0.0237
UGDH	UDP-glucose 6-dehydrogenase	-17.36	0.0002	0.0243
UQCC2	ubiquinol-cytochrome c reductase complex assembly factor 2	-18.91	6.70E-05	0.0237
MRPS24	URGCP-MRPS24 readthrough; mitochondrial ribosomal protein S24	-19.85	0.0007	0.0312
USMG5	up-regulated during skeletal muscle growth 5 homolog (mouse); microRNA	-5.82	0.0009	0.0346
USMG5P1	USMG5 pseudogene 1	-14.62	7.45E-05	0.0237
USP14	ubiquitin specific peptidase 14 (tRNA-guanine transglycosylase)	-10.86	0.0002	0.0247
USP3	ubiquitin specific peptidase 3	-11.66	0.0001	0.0237

USP7	ubiquitin specific peptidase 7 (herpes virus-associated)	-11.31	0.0004	0.0271
UTP18	UTP18 small subunit (SSU) processome component	-11.97	0.0003	0.0256
VAMP3	vesicle associated membrane protein 3	-12.96	0.0010	0.0347
VAMP4	vesicle associated membrane protein 4	-6.64	0.0004	0.0269
VAPB	VAMP (vesicle-associated membrane protein)-associated protein B and C	-7.53	0.0004	0.0272
VCAN	versican	-14.78	0.0006	0.0309
VDAC3	voltage-dependent anion channel 3	-26.25	0.0005	0.0291
VIM	vimentin	-23.71	0.0005	0.0289
VMP1	vacuole membrane protein 1; microRNA 21	-42.81	0.0002	0.0245
VPS25	vacuolar protein sorting 25 homolog (S. cerevisiae)	-8.42	0.0005	0.0285
VPS26A	VPS26 retromer complex component A	-11.04	0.0007	0.0322
VPS29	VPS29 retromer complex component	-5.81	0.0008	0.0330
VPS35	VPS35 retromer complex component	-21.05	5.41E-05	0.0237
WARS	tryptophanyl-tRNA synthetase	-26.69	0.0004	0.0269
WDR43	WD repeat domain 43	-12.44	0.0003	0.0255
WDR45B	WD repeat domain 45B	-11.68	0.0004	0.0269
WSB1	WD repeat and SOCS box containing 1	-12.66	0.0001	0.0237
WSB2	WD repeat and SOCS box containing 2	-14.18	0.0009	0.0333
XBP1	X-box binding protein 1	-10.24	0.0008	0.0331
XPO1	exportin 1	-15.23	0.0006	0.0309
XPOT	exportin, tRNA	-11.12	0.0009	0.0343
YAP1	Yes-associated protein 1	-7.62	0.0008	0.0331
YBX3	Y box binding protein 3	-26.32	0.0010	0.0351
YIF1A	Yip1 interacting factor homolog A (S. cerevisiae)	-7.03	0.0009	0.0333
YWHAB	tyrosine 3-monooxygenase/tryptophan 5-monooxygenase activation protein,	-32.16	0.0003	0.0261
YWHAH	tyrosine 3-monooxygenase/tryptophan 5-monooxygenase activation protein, eta	-15.38	0.0002	0.0238
ZBTB33	zinc finger and BTB domain containing 33	-6.79	0.0005	0.0277
ZDHHC2	zinc finger, DHHC-type containing 2	-5.76	0.0006	0.0309
ZDHHC3	zinc finger, DHHC-type containing 3	-10.14	0.0001	0.0237
ZFP36L1	ZFP36 ring finger protein-like 1	-9.98	0.0009	0.0343
ZFR	zinc finger RNA binding protein	-7	0.0004	0.0269
ZMPSTE24	zinc metallopeptidase STE24	-16.36	0.0003	0.0256
ZMYM4	zinc finger, MYM-type 4	-7.21	0.0005	0.0293
ZNF207	zinc finger protein 207; microRNA 632	-28.67	7.11E-05	0.0237
ZNF480	zinc finger protein 480	-9.64	0.0003	0.0256
ZNF528	zinc finger protein 528	-9.75	0.0003	0.0261
ZNF765	zinc finger protein 765; zinc finger protein 888; zinc finger protein 813	-9.61	0.0003	0.0255
ZZZ3	zinc finger, ZZ-type containing 3	-5.03	0.0010	0.0350
