# Supplementary material for: Platelet-activating factor acetyl hydrolase IB2 dysregulated cell proliferation in ovarian cancer
Source: Cancer Cell Int. 2021 Dec 20;21:697. doi: 10.1186/s12935-021-02406-9 (PMC8690939; doi:10.1186/s12935-021-02406-9)
Supplement: Supplementary file 4 — Additional file 4: Table S2. Top 20 representative enriched clusters by go biological processes. [file 12935_2021_2406_MOESM4_ESM.pdf]

Table S2. Top 20 clusters with their representative enriched go biological processes.

GO	Category	Description	Count	%	Log10(P)	Log10(q)
R-HSA-72766	Reactome Gene Sets	Translation	63	7.67	-35.62	-31.27
GO:0006412	GO Biological Processes	translation	94	11.45	-33.72	-29.66
WP3888	WikiPathways	VEGFA-VEGFR2 Signaling Pathway	65	7.92	-26.81	-23.05
GO:0044257	GO Biological Processes	cellular protein catabolic process	86	10.48	-25.07	-21.49
GO:0043312	GO Biological Processes	neutrophil degranulation	62	7.55	-21.85	-18.6
GO:0097190	GO Biological Processes	apoptotic signaling pathway	65	7.92	-19.64	-16.75
ko04141	KEGG Pathway	Protein processing in endoplasmic reticulum	34	4.14	-18.62	-15.82
R-HSA-5653656	Reactome Gene Sets	Vesicle-mediated transport	68	8.28	-18.29	-15.52
ko04520	KEGG Pathway	Adherens junction	23	2.8	-17.43	-14.75
R-HSA-1280215	Reactome Gene Sets	Cytokine Signaling in Immune system	67	8.16	-16.58	-13.99
GO:0034976	GO Biological Processes	response to endoplasmic reticulum stress	41	4.99	-15.87	-13.33
GO:0010256	GO Biological Processes	endomembrane system organization	48	5.85	-12.93	-10.51
R-HSA-194315	Reactome Gene Sets	Signaling by Rho GTPases	47	5.72	-12.75	-10.35
WP107	WikiPathways	Translation Factors	16	1.95	-12.33	-9.96
WP2864	WikiPathways	Apoptosis-related network due to altered Notch3 in ovarian cancer	16	1.95	-11.89	-9.55
ko05205	KEGG Pathway	Proteoglycans in cancer	29	3.53	-11.75	-9.43
R-HSA-8939211	Reactome Gene Sets	ESR-mediated signaling	30	3.65	-11.45	-9.17
R-HSA-109582	Reactome Gene Sets	Hemostasis	53	6.46	-11.45	-9.17
GO:0097435	GO Biological Processes	supramolecular fiber organization	57	6.94	-11.09	-8.83
GO:0051098	GO Biological Processes	regulation of binding	38	4.63	-10.94	-8.68
